# Structure and assembly of the S-layer in *C. difficile*

**DOI:** 10.1038/s41467-022-28196-w

**Published:** 2022-02-25

**Authors:** Paola Lanzoni-Mangutchi, Oishik Banerji, Jason Wilson, Anna Barwinska-Sendra, Joseph A. Kirk, Filipa Vaz, Shauna O’Beirne, Arnaud Baslé, Kamel El Omari, Armin Wagner, Neil F. Fairweather, Gillian R. Douce, Per A. Bullough, Robert P. Fagan, Paula S. Salgado

**Affiliations:** 1 Biosciences Institute, Faculty of Medical Sciences, Newcastle University, Newcastle upon Tyne, UK; 2 Krebs Institute, School of Biosciences, University of Sheffield, Sheffield, UK; 3 Florey Institute, School of Biosciences, University of Sheffield, Sheffield, UK; 4 Institute of Infection, Immunity and Inflammation, College of Medical, Veterinary and Life Sciences, University of Glasgow, Glasgow, UK; 5 Diamond Light Source, Oxfordshire, UK; 6 Department of Life Sciences, Imperial College London, London, UK; 7 Present Address: Royal Society of Chemistry, Burlington House, Piccadilly, London, UK; 8 Present Address: Department of Immunology, Oslo University Hospital, Oslo, Norway

**Keywords:** Electron microscopy, X-ray crystallography, Bacterial structural biology, Cellular microbiology

## Abstract

Many bacteria and archaea possess a two-dimensional protein array, or S-layer, that covers the cell surface and plays crucial roles in cell physiology. Here, we report the crystal structure of SlpA, the main S-layer protein of the bacterial pathogen *Clostridioides difficile*, and use electron microscopy to study S-layer organisation and assembly. The SlpA crystal lattice mimics S-layer assembly in the cell, through tiling of triangular prisms above the cell wall, interlocked by distinct ridges facing the environment. Strikingly, the array is very compact, with pores of only ~10 Å in diameter, compared to other S-layers (30–100 Å). The surface-exposed flexible ridges are partially dispensable for overall structure and assembly, although a mutant lacking this region becomes susceptible to lysozyme, an important molecule in host defence. Thus, our work gives insights into S-layer organisation and provides a basis for development of *C. difficile*-specific therapeutics.

## Abstract (web summary)

The S-layer is a two-dimensional protein array that covers the cell surface of many bacteria and archaea. Here, the authors use high-resolution X-ray crystallography and electron microscopy to provide detailed insights into S-layer organisation and assembly for the bacterial pathogen *Clostridioides difficile*.

## Introduction

The surfaces of most bacteria and archaea are covered with a proteinaceous coat, the surface or S-layer, which is formed through the self-assembly of individual protein subunits into a regularly spaced, two-dimensional array^1^. The tendency of S-layer proteins to spontaneously form 2D assemblies has hampered structure determination and restricted understanding of both their function and architecture. To date, all the S-layer proteins studied in detail have a two-domain organisation, where the assembly domain is responsible for the formation of the paracrystalline layer, and the anchoring domain allows attachment of the S-layer to the cell wall^1^. Structures of S-layer assembly domains determined from a limited number of species^2–6^ showcase the huge diversity of S-layer proteins and their arrangements, and provide insights into mechanisms of anchoring and structural organisation. However, a complete, intact atomic-level structure of both the assembly and anchoring domains has not yet been presented for any major S-layer protein.

The ubiquity of the S-layer and its function as a physical barrier at the host-pathogen interface renders the S-layer a very important target in the search for antimicrobials effective against medically important bacteria, such as *Clostridioides difficile*. The Gram-positive opportunistic pathogen, *C. difficile*, is the leading cause of hospital-acquired, antibiotic-associated diarrheal disease globally^7^. *C. difficile* infection (CDI) causes substantial morbidity and mortality with severe disease characterised by intestinal inflammation, resulting in extensive damage to the colon^7^. This pathology has largely been attributed to the action of potent toxins that disrupt the cytoskeletal structure and the tight junctions of target cells causing rounding and ultimately death^8^. Toxins also initiate a proinflammatory response via activation of the inflammasome^9^. Other less studied factors associated with inflammation, including the S-layer, contribute to the recruitment of neutrophils, via a TLR/Myd88 dependent signalling pathway^10,11^. However, the exact role of the S-layer in pathogenesis remains unclear.

**Fig. 1:**
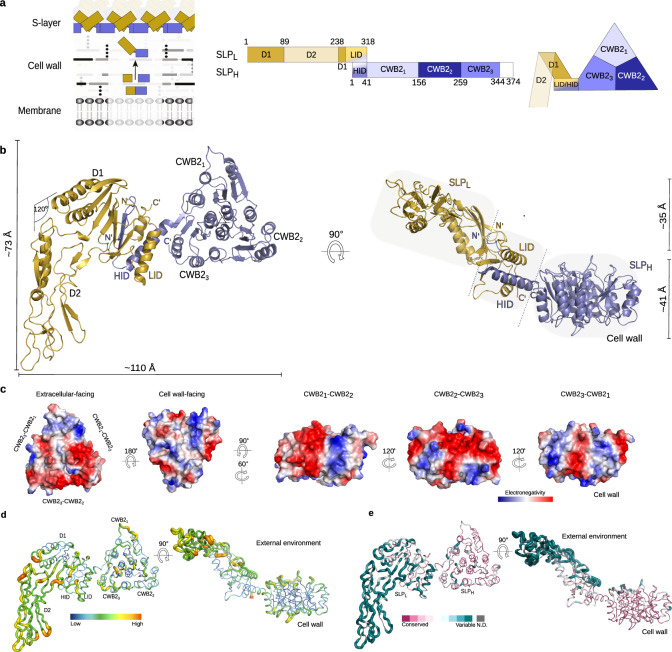
Architecture of *C. difficile* SLP_H_/SLP_L_ (H/L) complex. **a** SlpA arrangement on the cell surface (left; SLP_L_ coloured in gold and SLP_H_ in slate blue) with detailed organisation of protein building blocks in its primary sequence (middle) and quaternary structure (right). Numbering based on the subunits of SlpA from strain CD630, S-layer cassette type 7 (SLCT-7), PBD ID: 7ACY. **b** Cartoon representation of H/L complex as viewed from the external environment (top view, left) and side (right). The SLP_L_ protrudes above the SLP_H_ subunit, creating a two-plane arrangement. Three distinct structural features are observed: SLP_H_, D1 and D2, and LID/HID (regions highlighted in grey). **c** Charge distribution across CWB2 motifs in SLP_H_ as Poisson–Boltzmann electrostatic potential calculated for SlpA_CD630_, shown as a gradient (positive in blue to negative in red). Views are shown from the extracellular and cell wall surfaces, followed by side views of the lateral faces defined by two interacting CWB2s. **d** Putty representations of SlpA_CD630_ H/L complex showing B-factors ranging from low (blue and narrow) to high (red and wide). High B-factors are indicative of disorder/flexible regions. **e** Conservation of the SlpA sequence across annotated SlpA cassette types (SLCTs) depicted on putty representations of SlpA_CD630_ H/L complex, coloured from conserved (purple) to variable (cyan). Conservation was calculated using Consurf web server.

In *C. difficile*, the S-layer largely consists of a major S-layer protein, SlpA (Fig. 1a), which assembles into a paracrystalline array enveloping the cell. Considerable variation is observed between different *C. difficile* strains, with 13 different S-layer cassette types (SLCTs) identified to date^12^. Accessory components of the S-layer, belonging to a family of 28 cell wall proteins (CWPs), are inserted in the proteinaceous envelope, and make up an estimated 5–20% of the S-layer, providing additional functions^1^. We previously reported that an S-layer-null mutant of *C. difficile* was avirulent in the hamster model of acute disease, despite apparent normal colonisation of the caecum and colon^13^. Notably, absence of a functional SlpA resulted in a range of pleiotropic effects, including reduced toxin production. Although this work suggested a role for S-layer in *C. difficile* disease, reduced toxin expression made it impossible to establish a direct functional role.

To fully interrogate the role of the S-layer in pathogen survival and host disease severity, a complete, high-resolution S-layer structure is essential. Moreover, the uniqueness of S-layers across different bacteria makes these arrays attractive targets for species-specific therapeutic interventions, provided sufficient structural and functional data are available.

Here we present the complete atomic-level model of the assembly and anchoring domains of the S-layer in *C. difficile*, generated by combining high-resolution X-ray crystallography with electron microscopy.

## Results

### *C. difficile* major S-layer component forms an intricate complex

The major protein of the *C. difficile* S-layer, SlpA, is post-translationally cleaved into two S-layer proteins (SLPs): the high molecular weight (HMW) and low molecular weight (LMW)^14^, herein referred to as SLP_H_ and SLP_L_, respectively. These subunits then form a complex (referred to as H/L) that is incorporated in the S-layer (Fig. 1a). Unlike in other S-layer structures determined to date, in *C. difficile*, the anchoring domain, responsible for linking the S-layer to the cell wall, and the assembly domain, which forms the 2D paracrystalline array, are one and the same, both proposed to be present in SLP_H_^15,16^.

The structures of the full-length H/L complexes of representatives of two SLCTs (SLCT7, strain CD630 and SLCT7b, strain R7404) were determined using X-ray crystallography; we combined single anomalous dispersion sulfur data (S-SAD) and molecular replacement using substructures of the interacting domains (SLP_L_ interacting domain, LID, and SLP_H_ interacting domain, HID; PDB ID: 7ACW) and SLP_L_/HID (PDB ID: 7ACV). Final models to a resolution of 2.55 Å (PDB ID: 7ACY) and 2.65 Å (PDB ID: 7ACX) for SlpA_CD630_ and SlpA_R7404_, respectively, reveal a nearly identical fold (RMSD over aligned Cα – core – 2.1 Å) and here we focus our analysis on the best overall model, SlpA_CD630_. Our H/L structural model reveals three distinct regions: a pseudo-threefold symmetric SLP_H_ tile, an intricate LID/HID interacting motif and a third region composed of two domains, D1 and D2, of SLP_L_ (Fig. 1b). These regions define two separate planes, with the SLP_L_ spanning ~35 Å above the SLP_H_ plane, linked by the LID/HID motif (Fig. 1b).

SLP_H_ is composed of three sequence-conserved cell wall binding motifs 2 (CWB2), which define the *C. difficile* cell wall protein (CWP) family^1^, and the HID. The three CWB2 motifs form a triangular prism and adopt an intertwined fold, with a β-strand from one CWB2 inserting into the neighbouring domain to complete a β-sheet (β2_H_ in CWB2_1_ completes CWB2_3_, strand β6_H_ from CWB2_2_ inserts into CWB2_1_ and β10_H_ in CWB2_3_ is inserted into CWB2_2_) sandwiched between two α-helical regions (Supplementary Fig. 1). At the core of the tile sits a helical bundle with each individual CWB2 contributing one α-helix (CWB2_1_ - α3_H_, CWB2_1_ - α7_H_, CWB2_1_ - α11_H_) while two α-helices define the vertices of the pseudo-threefold arrangement (Supplementary Fig. 1). This trimeric arrangement generates a distinctive charge distribution across each prism face, creating surfaces with complementary charges (Fig. 1c). The pseudo-threefold organisation of the CWB2s is conserved in the structures of two minor constituents of the S-layer, Cwp6 and Cwp8 (Supplementary Fig. 2)^17^. The environment- and cell-facing sides of SLP_H_ exhibit considerable charge differences, with a mostly negatively charged external surface and a largely non-polar cell wall-facing base, decorated by positive patches (Fig. 1c). The positive patches at the cell-wall base could provide the mechanism for anchoring SlpA to the cell wall via interactions with the anionic secondary cell wall polymer PSII^16^.

It is worth noting that, despite the lack of homology between S-layer proteins from different species, all anchoring domains determined to date have a predominantly α-helical structure^18,19^, which suggests that helical bundles might be a favoured organisation for S-layer attachment to the cell. Notably, the S-layer homology domain (SLH) responsible for anchoring the S-layer in *Bacillus anthracis* has a central three α-helical bundle^19^ similar to the core of the CWB2 triad organisation in SLP_H_.

**Fig. 2:**
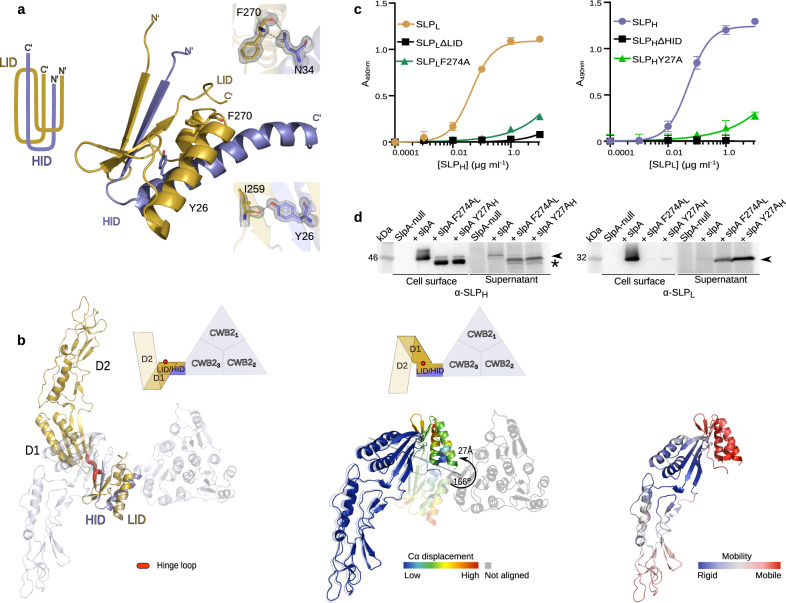
Interactions and flexibility in *C. difficile* SLP_H_/SLP_L_ (H/L) complex. **a** Paperclip organisation of the interacting domains LID/HID is maintained by a range of interactions, with selected interface residues identified in strain R7404 (SLCT-7b, PDB ID: 7ACW) depicted as sticks. 2m*Fo-*D*Fc* electron density map is shown on the interacting amino acid pairs as a grey mesh contoured at 1.5 σ. Specific interatomic interactions identified with PDBePISA are represented as a dashed line. **b** Superimposing structures of SLP_L_/HID (gold/slate blue, PDB ID: 7ACV) onto the native complex of SlpA_R7404_ (SLCT-7b, PDB ID: 7ACX) (blue/white) reveals the flexibility of the LID-D1 linker, as illustrated by rotation of D1-D2 domains in relation to fixed position of LID/HID motif (left). The hinge loop enabling this conformational flexibility (determined by DynDom6D) is coloured in red. The backbone displacement (coloured from blue – low, to red – high Cα displacement deviation) is shown on the alignment of D1-D2 region of both structures (middle; SLP_L_/HID – opaque, H/L – semi-transparent) with the rotation angle of the LID/HID motif indicated with an arrow. Structural dynamics (right) of SLP_L_/HID, represented as increasing mobility (coloured blue – rigid, to red - mobile), calculated based on elastic network models implemented in DynOmics ENM version 1.0 server. **c** Probing of CD630 H/L complex interactions in vitro with ELISA, comparing effects of intact SLP_L_ (gold circles), SLP_H_ (slate blue circles), variants lacking interacting domains (black squares) and substitution mutants of F274A (structurally equivalent to F270 in R7404 LID/HID depicted in **c**, dark green triangles) and Y27A (structurally equivalent to Y26 in R7404 LID/HID in **c**, light green triangles) on H/L complex formation. Graphs represent mean ± standard deviation (SD) of *n* = 3 experiments, with least-squares curve fit of product formed upon the interaction of the two subunits. Source data provided in Source data file. **d** Western blot of cell surface extracts and culture supernatants, detecting (black arrowhead) SLP_H_ (left) and SLP_L_ (right) in strains devoid of endogenous *slpA* and expressing plasmid-borne SlpA_CD630_ native protein or variants with either F274A_L_ or Y27A_H_ substitution mutants in SLP_L_ or SLP_H_ (denoted in subscript), respectively (n = 1). Detected product of partial degradation of SLP_H_ indicated with an asterisk. Source data provided in Source Data file.

The HID motif interlocks with LID in an arrangement reminiscent of a paperclip: LID α-helices α4_L_ and α5_L_ and HID α1_H_ pack against a β-sheet formed by insertion of β1_H_ from HID between LID β-strands, β18_L_ and β21_L_ (Fig. 2a and Supplementary Fig. 1). This novel structural motif locks SLP_L_ and SLP_H_ together, providing a structural basis for the stability of the H/L complex^15^.

To identify residues essential for the interaction of the SLP_L_ and SLP_H_ subunits, we analyzed H/L complex formation in an enzyme-linked immunosorbent assay (ELISA) with a panel of individual point mutants (Fig. 2c and Supplementary Fig. 3). Mutations of a single amino acid within LID (F274A) or HID (Y27A) in SlpA_CD630_ were sufficient to destabilise the H/L complex (Fig. 2c). Moreover, expression of either point mutant in an SlpA-null background resulted in SLP_L_ shedding from the cell surface of *C. difficile* and detection of a fraction of SLP_H_ in the culture supernatant. Loss of SLP_L_ also resulted in partial degradation of SLP_H_ (Fig. 2d): N-terminal sequencing revealed truncation of the HID, indicating that this region is unstable in the absence of the LID/HID interaction (Fig. 2d).

SLP_L_ protrudes from the interacting domains, with D1 closest to the SLP_H_ plane and D2 extending outwards at an angle of ~120°, away from the long axis of D1. As we previously described^15^, whilst D1 is well ordered, formed by a 5-strand β-sheet packed against two α-helices (Supplementary Fig. 1), D2 is predominantly composed of long, flexible loops, particularly at the externally exposed surface (Fig. 1b). This domain is characterised by high B-factors (Fig. 1d) and weaker electron density. The structural flexibility accommodates the high sequence variability observed across SLCTs (Supplementary Fig. 4) - residues with high B-factor map almost perfectly to sequence variation hotspots in D2 (Fig. 1d, e).

Available structures of assembly domains of other S-layers show a predominance of β-strand rich domains, organised to create the 2D array^2,3,5,18^. The S-layer assembly domains from two Bacilli^2,5,18^ rely on multiple structurally related β-sandwich protomers. These form a central tile-like body, with an arm, composed of the more external protomers, extending outwards and providing both extra lattice contacts as well as a certain degree of flexibility^5^. In *C. difficile*, the β-strand rich D1 and D2 domains also extend outwards from the SLP_H_ tiles and seem to adopt a similar confirmation; even though they are not thought to be part of the core assembly domain, they could thus also contribute to S-layer assembly contacts.

Conformational flexibility in the organisation of SlpA is further demonstrated by different arrangements observed in the structure of a truncated derivative of SlpA (SLP_L_/HID) and the H/L complex. In the structure of the SLP_L_/HID complex (PDB ID: 7ACV), the D1-D2 domains exhibit an orientation relative to the interacting domains, which is different from that seen in the corresponding H/L complex (R7404 strain, SLCT-7b, PDB ID: 7ACX). Our models indicate the presence of a hinge, formed by the D1-LID linker (Fig. 2b). Analysis of the architecture and simulated motions (DynDom6D v1.0) in these models identified regions of D1-D2 and LID/HID as two dynamic domains, with the residues making up the linker between the D1 C-terminal β-strand (β17_L_) and the LID N-terminal β-strand (β18_L_) acting as an interdomain hinge (Fig. 2b). The calculated rotation angle of the centres of mass of the D1-D2 region relative to LID/HID of 166° suggests a high degree of flexibility of these regions, at least in the absence of the CWB2-containing SLP_H_ core, with minimal effects on the fold of each individual domain. The fact that we observed this conformational flexibility in our protein crystal models, with no apparent effect on the fold of individual regions, suggests how the effector domains of other CWPs inserted in the functional S-layer can be accommodated by flexible rearrangement of D2. Interestingly, despite the overall similarity between our models, different SLPs can adopt alternative conformations: in SlpA_R7404_ (PDB ID: 7ACW), one SLP_L_ molecule packs away from SLP_H_ when compared to SlpA_CD630_ (PDB ID: 7ACY) due to a rotation of 9° around the LID/HID axis, further strengthening the idea that D1-D2 and D1-LID linkers provide flexibility to the structure. This flexibility among the most external domains, with certain loops acting as hinges for flexible conformation of parts of the structure, has also been described in other known S-layer structures^18^ and could have a functional role in S-layer assembly.

### Crystal lattice reflects in situ S-layer assembly

**Fig. 3:**
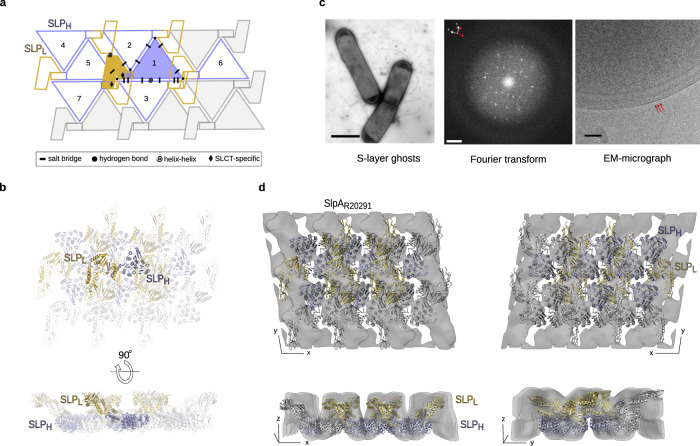
Planar crystal packing in the X-ray structure fits the in situ packing of the native S-layer. **a** 2D schematic of H/L complex crystal packing, indicating the interaction network linking a single H/L (slate blue/gold) complex with six other molecules in a planar arrangement generated by SLP_H_ tiling. Array is depicted as seen from the extracellular environment, with symbols representing key interaction types in the crystal lattice, detailed in Supplementary Fig. 4. **b** Cartoon representation of the H/L planar array (PDB ID 7ACY, coloured as in **a**, views as defined in Fig. 1b). **c** Native *C. difficile* S-layer ghosts (electron micrograph, negatively stained, left. Scale bar: 2 µm) were used to compute Fourier transforms (middle). Micrograph is a lower magnification (×3000) of ghosts used to collect the images used (82351x) for Fourier transform computation and is representative of the morphology of S-layer ghosts. Typically spots from two or more lattices were observed. Fourier transform is representative of 36 images collected for R20291. Reciprocal lattice axes (red and white axes) are indicated for two observed lattices (scale bar 0.0125 Å^−1^). Intact frozen-hydrated *C. difficile* cells, examined by cryo-electron microscopy (right), show distinctive ridged surface indicated by red arrows (scale bar 50 nm). Micrograph is representative of whole-cell images, which were not used for data analysis and 2D map calculations. Instead, these were computed from homogenised fragments, as described in the Methods section. **d**, Orthogonal views of the 3D reconstruction of negatively stained S-layer ghost indicating the overall envelope in the native lattice. A rigid body fit of the structure of H/L complex determined by X-ray crystallography (PDB ID: 7ACY, cartoon representation, SLP_L_ - gold, SLP_H_ - slate) indicates a similar arrangement in the native S-layer ghosts and crystal packing. Reconstruction is shown from the environment (top left) and cell wall (top right), and side views in the 2D plane (bottom panels).

Due to the propensity of S-layer proteins to spontaneously self-assemble into 2D arrays^20^, we hypothesised that the packing of our crystal structures might be reflective of native S-layer assembly. In the crystal, two H/L complexes, related by pseudo-twofold symmetry, are present in the P1 asymmetric unit, packed in a 2D planar array (Fig. 3) which is then stacked to extend the crystal into the third dimension. The 2D lattice is achieved by tiling of SLP_H_, with interlocked ridges of SLP_L_ molecules covering gaps between the tiles, creating a tightly packed layer (Fig. 3a, b). Lattice contacts between CWB2 motifs of neighbouring SLP_H_ molecules involve helix-helix interactions between the symmetry-related copies of α12_H_ (see topology in Supplementary Fig. 1), as well as electrostatic interactions generating a tightly bonded network (Fig. 3a and Supplementary Fig. 5a).

We investigated if the planar crystal packing observed in the X-ray structure reflects the in situ packing of the native S-layer assembly. Intact S-layer extracted from *C. difficile* vegetative cells formed collapsed capsules exactly mimicking the size and shape of the originating cell (native S-layer ghosts). These double-layered 2D crystals were interrogated by electron crystallography, with rotationally separated diffraction patterns observed from images of the superimposed layers (Fig. 3c). As hypothesised, the *p*2 symmetric 2D lattices of native S-layer ghosts (*a* = *b* = 80 Å, *γ* = 100°) were consistent with unit cell parameters of the stacked lattices in the 2D plane of the X-ray crystals (*b* = 78 Å, *c* = 80 Å, *α* = 100°) (Supplementary Tables 1–3), pointing towards a similar packing (Fig. 3c). The 3D reconstruction from images of the native ghosts revealed a molecular envelope with a staggered ridged surface on one face of the S-layer, with deep grooves between the parallel ridges, and an opposing surface defined by paired, globular domains arranged in rows (Fig. 3d). These features recapitulate the surface characteristics of the H/L array in the X-ray crystal structure. Indeed, manual fitting of the 2D X-ray lattice as a single rigid body into the EM density matches the ridged surface to SLP_L_, with the paired globular domains on the opposite face corresponding to the SLP_H_ CWB2 motifs (Fig. 3d). The ridges are also observed in cryo-electron microscopy (cryo-EM) side views of intact cells (Fig. 3c). This confirms that the X-ray crystal lattice of the H/L complex has the same overall arrangement as the in situ lattice of a mature S-layer in intact cells, therefore establishing our crystallographic model as a template for S-layer assembly in *C. difficile*.

### Principles of S-layer assembly

Analysis of crystal packing of the H/L complex revealed key principles governing S-layer assembly. The charge distribution generated by the trimeric arrangement of the CWB2s provides complementary charges across the lateral faces of the SLP_H_ triangular prism tile (Fig. 1c), allowing these interactions to be established (Fig. 3a and Supplementary Fig. 5a). The structural analysis of homology models of other SLCTs suggests that most of the interactions between neighbouring SLP_H_ molecules, which define those interfaces, are conserved across different SLCTs (Supplementary Fig. 5b), indicating that they are likely to be important for *C. difficile* S-layer assembly. It is worth noting that the most conserved interactions are at the interface of neighbouring CWB2_3_-CWB2_1_ motifs but also involve residues from SLP_L_ (Fig. 3a and Supplementary Fig. 5b, top 6 rows). This conservation suggests that SLP_L_ is also important for maintaining S-layer packing, together with the CWB2 assembly motifs, and that targeting these interactions could lead to disruption of the array.

Our analysis of the charge distribution of the CWB2 motifs in Cwp6 and Cwp8 (Supplementary Fig. 2) indicates that the charge complementarity between the H/L complex and these minor S-layer components would also be possible. However, homology between CWPs and SlpA is restricted to the CWB2 trimeric motif as CWPs have distinct accessory domains, replacing the SLP_L_^1^. These structurally diverse domains are presumably accommodated in the S-layer whilst maintaining the integrity of the crystalline array. Based on the observation that most of the X-ray crystallographic model of the H/L complex fits well into the envelope defined by the EM reconstruction except for part of the D2 domain, we propose that this region of SLP_L_ might confer further flexibility to the assembled S-layer. It is possible that D2 adopts a slightly different position relative to SLP_H_ in the mature S-layer as it could be better accommodated by a rotation of 11° relative to D1 into the EM envelope (Supplementary Fig. 6). It is worth noting that this slight reorientation of D2 does not alter the overall packing of H/L complex into the 2D array (Supplementary Fig. 6). The strikingly different arrangements of the D1-D2 domains in SLP_L,_ relative to the interacting domains observed in the H/L and SLP_L_/HID structures (Fig. 2a), are consistent with this proposed flexibility.

While increasing numbers of S-layer structural models are available^2–6,18,21^, to our knowledge, a complete X-ray structure of a major S-layer protein where the crystal lattice mimics S-layer assembly on the cell has not been previously reported. This indicates that S-layer assembly in *C. difficile* does not require an underlying ordered polysaccharide array, unlike in LPS-mediated S-layer anchoring in the Gram-negative *C. crescentus*^6^. As the S-layer in *C. difficile* is anchored via interactions of the CWB2 motifs with PSII^16^, a much simpler glycan unlikely to be ordered at the cell surface, it is not surprising that the protein can assemble independently. To elucidate the anchoring mechanisms of *C. difficile* S-layer, we are investigating the interactions of SLP_H_ and the H/L complex with PSII using a combination of biochemical, biophysical and structural methods.

### Probing the S-layer assembly model

**Fig. 4:**
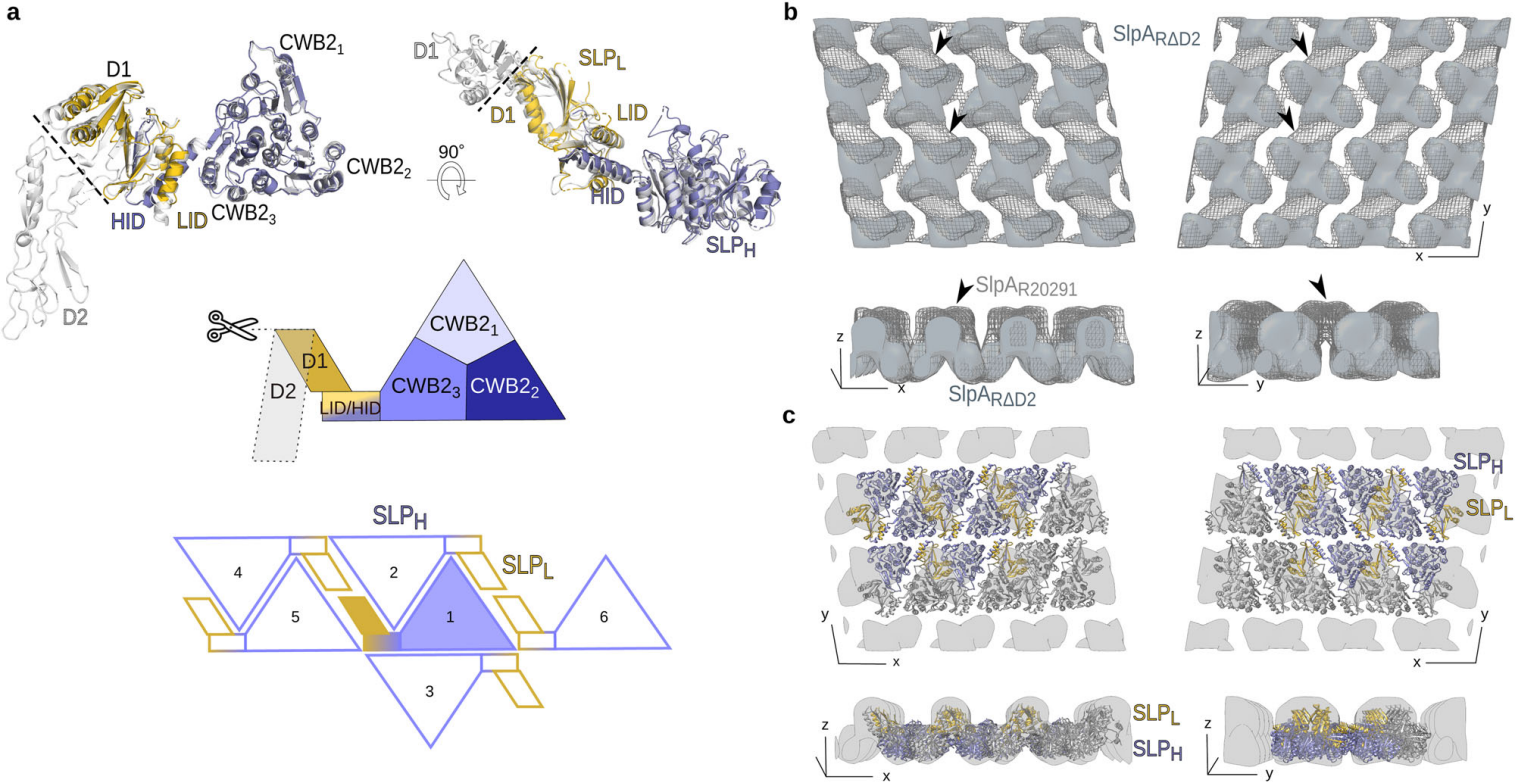
The flexible D2 domain is dispensable for S-layer assembly. **a** Cartoon representation of the SlpA_RΔD2_ H/L complex crystal structure (slate blue and gold, PDB ID: 7ACZ), superimposed onto SlpA_CD630_ H/L complex structure (PDB ID: 7ACY, grey). Deleted D2 region is marked with a dashed line on the CD630 structure and corresponding schematic representation of the complex. Views as in Fig. 1b. 2D schematic of H/L complex crystal packing in SlpA_RΔD2_, indicating the interaction network linking a single H/L (slate blue/gold) complex with five other molecules in a planar arrangement generated by SLP_H_ tiling. **b** Superimposition of the 3D reconstruction of negatively stained S-layer ghost containing SlpA devoid of domain D2 (SlpA_RΔD2_, light blue solid surface) on the reconstruction of native wild-type S-layer ghost (SlpA_R20291_, grey mesh). The missing density can be largely ascribed to that of the missing D2 domain (indicated with black arrowheads). Views as in Fig. 2d. **c** Fit of the SlpA_RΔD2_ structure determined by X-ray crystallography (coloured as in **c**) into the S-layer (grey) reconstruction indicates a similar arrangement in the crystal packing and the native array. Views as in **b**.

To further test our S-layer assembly model, we sought to generate a mutated S-layer that still assembles into a paracrystalline array. The observed interactions for assembly involve mainly the SLP_H_ tiles and D1 in SLP_L_. Moreover, these regions are conserved across different SLCTs (Supplementary Fig. 5b). We therefore hypothesised that the structurally flexible and less conserved D2 domain might be dispensable for maintaining S-layer assembly and engineered a mutant strain devoid of D2, named RΔD2 (producing SlpA_RΔD2_). The X-ray crystal structure of the resulting H/L complex (Fig. 4a, PDB ID: 7ACZ) superimposes readily onto the full-length SlpA_CD630_ model (core RMSD 1.5 Å), with the absence of D2 not perturbing the overall protein fold. Moreover, the crystal lattice is similar to wild type, with equivalent interactions between SLP_H_ tiles and D1 domains (Supplementary Fig. 7).

Analysis of native S-layer ghosts from bacteria producing the SlpA_RΔD2_ revealed that, despite lacking nearly half of SLP_L_ (145 of 318 amino acids), an S-layer with identical lattice parameters to the wild type is still formed. The reconstructed molecular envelope retains the paired globular domain organisation (Fig. 4b, c), but the outer surface lacks the staggered ridge feature seen in the wild-type EM reconstruction (Fig. 4b, c), confirming our assignment of this density to the D2 domain. This is further validated by difference maps of cryo-EM projections for full-length SlpA_R20291_ and SlpA_RΔD2,_ which show a region of significant difference density, matching the position of D2 in the complete structure (Supplementary Fig. 8).

Together, the structural models of SlpA_RΔD2_ and the corresponding S-layer reconstruction confirm our model for S-layer assembly, where SLP_H_ tiling and SLP_L_ D1 domains provide the key contacts for paracrystalline array formation.

### Permeability of the S-layer

**Fig. 5:**
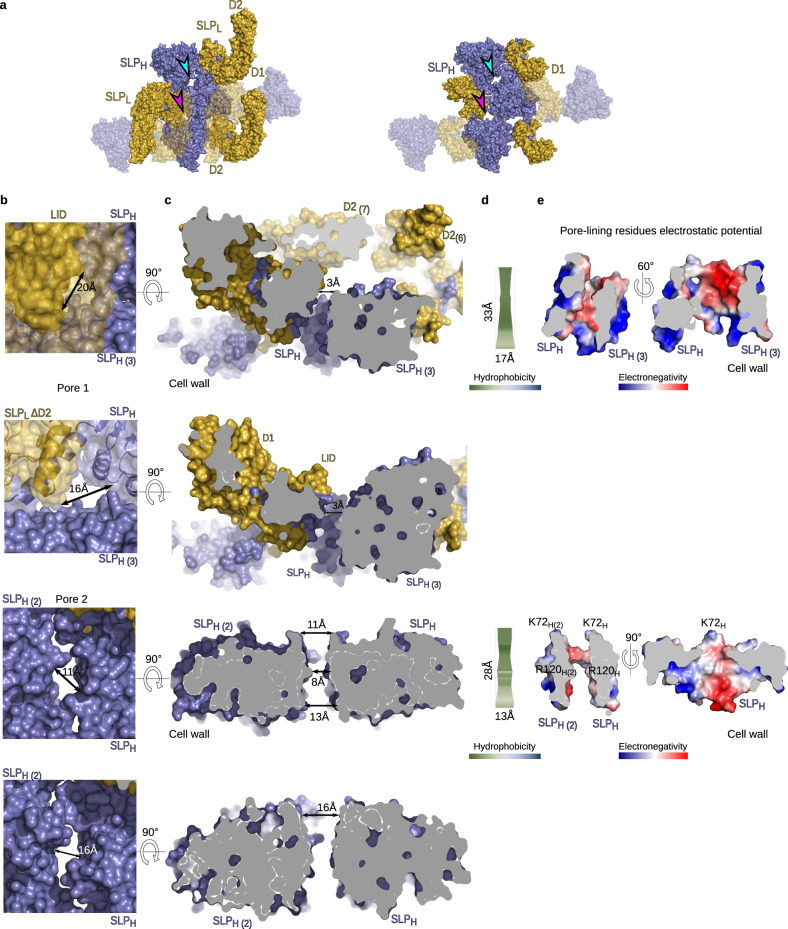
*C. difficile* S-layer is a tightly packed array with very narrow pores. **a** Surface representation of wild-type H/L (7ACY, left) and SlpA_RΔD2_ H/L (7ACZ, right) crystal packing showing pores in the 3D crystal lattice. Positions of pores marked with arrowheads (pore 1 in magenta, pore 2 in cyan) are equivalent in both lattices. **b** Zoomed-in view of the pores generated by H/L multimerization. Pore 1, top view covered by D2 in SlpA_CD630_ (first panel) and in SlpA_RΔD2_ (second panel). Pore 2 – top view for SlpA_CD630_ (third panel) and SlpA_RΔD2_ (fourth panel). Widest openings are labelled for each pore. Arrows indicate the widest points in each pore, which are exposed in SlpA_RΔD2_ due to the lack of D2 that completely covers it in SlpA_CD630_. **c** Cross-section views of pore 1 and pore 2 in SlpA_CD630_ (first and third panels, respectively) and SlpA_RΔD2_ (second and fourth panels, respectively). Neighbouring SLP_H_ (slate blue) and SLP_L_ (gold) molecules that create the pores are shown in surface representation. **d** Hydrophobicity characteristics of the residues lining pore 1 (top) and 2 (bottom) calculated in ChexVis (see Methods section for details) according to Kyte-Doolittle scale, ranging from hydrophilic (green) to hydrophobic (blue), as per hydrophobicity gradient key. **e** Poisson–Boltzmann electrostatic potential calculated for residues lining pore 1 (first panel) and 2 (second panel) in SlpA_CD630_ represented as a charge distribution (positive in blue and negative in red, as per electronegativity gradient key). Views and scale are as in **c** (left) and as a slice across the largest pore surface (right). Pseudo-symmetry-related lysine residues at the top and arginine residues at the bottleneck of pore 2 are highlighted.

S-layers have been proposed to act as a molecular filter^22^ but given the tight packing of the SlpA lattice, the bulk of the *C. difficile* cell surface seems virtually impenetrable to large molecules; this is despite being able to secrete toxin in the apparent absence of cell lysis^23^. Other S-layer proteins have been proposed to create relatively permeable arrays^24,25^, with pores ranging from ~30 Å up to 100 Å in diameter and possibly wider^3,6,25^. In contrast, tiling of SLP_H_ and the SLP_L_ ridges generate a compact lattice apart from two distinct pores in the *C. difficile* S-layer array. Pore 1 (Fig. 5a, cyan arrow, between molecules 1 and 3) is ~20 Å across the widest point at the environment-facing surface but is partially occluded by the LID/HID motif narrowing it down to an 11 Å wide cavity. The interlocked D2 domains of adjacent SLP_L_ molecules cap this pore, further reducing access from the external environment to the cell wall (Fig. 5b–e, top). The second pore, formed between two SLP_H_ (Fig. 5a, molecules 1 and 2) is fully accessible from both outer and inner surfaces of the layer. It has a width of approximately 11 Å (pore 2, Fig. 5a, pink arrow), but is narrowed to 8 Å by two pseudo-symmetry equivalent arginine residues within 10 Å of the pore outward side (Fig. 5b–e, bottom).

Importantly, the absence of D2 exposes pore 1 between SLP_H_ tiles (Fig. 5b–e, middle), which is occluded by interlocking D2 domains in the full-length structure. This creates two openings in the array of about 16 Å, which could indicate a more permeable S-layer than in the wild-type structure, with twice as many pores, of slightly increased size. Moreover, many of the residues lining the two exposed pores in this lattice are not resolved in the electron density of the SlpA_RΔD2_ and could not be modelled, suggesting higher flexibility and, therefore, potentially weaker interactions.

The lining of the pores observed in the crystal lattices (Fig. 3) are highly hydrophilic (Fig. 5d), suggesting that only small, hydrated ions could easily diffuse into the cell. Pore 1, mostly occluded by D2 in the full H/L complex, has a mixed charged distribution, with patches of both positive and negative charges throughout (Fig. 5b–e). In contrast, the fully exposed pore 2 is mostly negatively charged, indicating that positively charged small metabolites could preferentially diffuse via this pore. The electropositive patch formed by two pseudo symmetry-related lysines covering the outermost opening of this pore and the two arginines at the innermost side (Fig. 5e) could provide some restricted access for charged species.

**Fig. 6:**
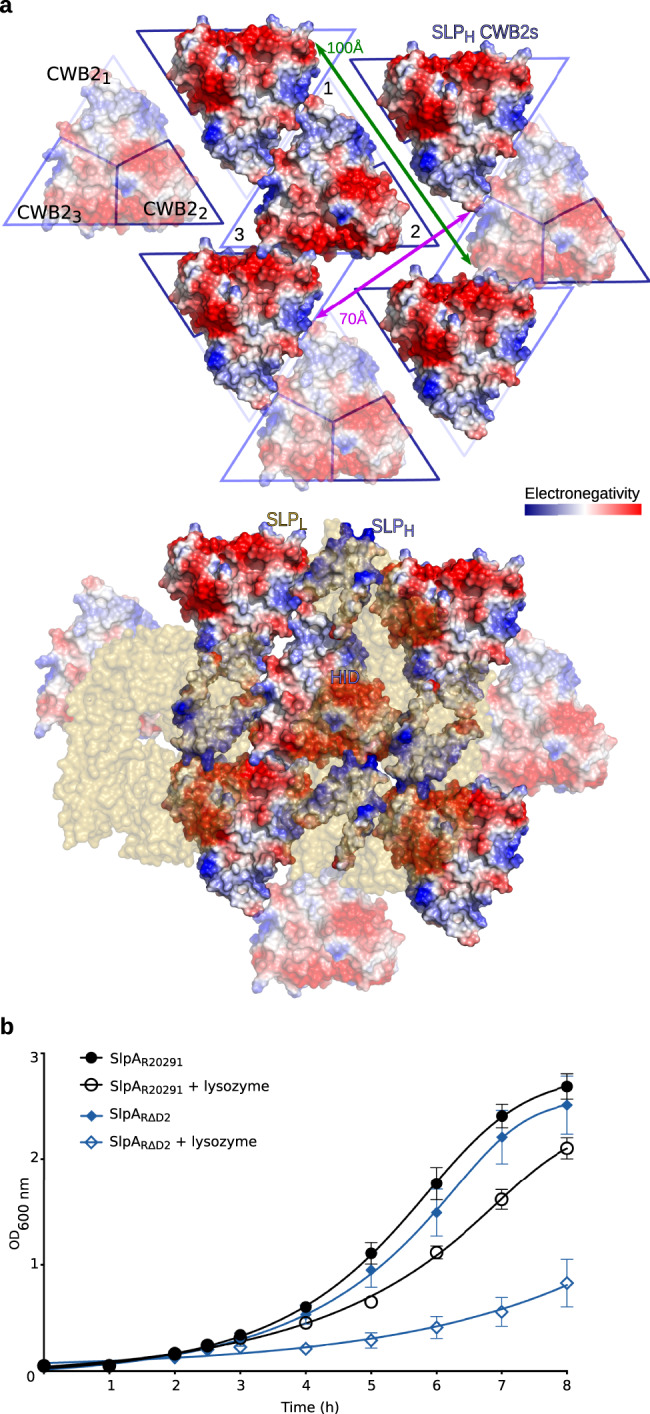
SLP_H_ and SLP_L_ create a tightly packed 2D array. **a** Tiling of SLP_H_ CWB2 motifs via charge complementarity across each triangular prism face. Poisson–Boltzmann electrostatic potential calculated for SlpA_CD630_ SLP_H_, represented as a charge distribution (positive – blue; negative - red) on the surface representation of SLP_H_ array. Interacting surfaces between molecules 1–2, defined by pseudo-symmetry related CWB2_3_-CWB2_1_, and between molecules 1–3, defined by symmetry-related CWB2_3_ triangular prism faces, are labelled. Cavity between symmetry-related CWB2_1_-CWB2_2_ surfaces, represented by green arrows (top) is partially obstructed by HID domains (electrostatic potential surface representation) and completely occluded by SLP_L_ (gold) as shown on the bottom panel. A long cavity of ~70 Å at the CWB2_2_ vertices represented by purple arrow (top) is also occluded by HID domains and interacting SLP_L_ molecules (bottom). **b** Lysozyme resistance. Cultures of SlpA_R20291_ (circles) and SlpA_RΔD2_ (diamond) were inoculated at an OD_600nm_ of 0.05 and grown anaerobically at 37 °C with hourly OD_600nm_ measurements. Where indicated (open circles, SlpA_R20291_; open diamonds, SlpA_RΔD2_) lysozyme (500 μg ml^−1^) was added after 2.5 h growth. Data are presented as mean values (±SD) from two biological replicates, assayed in triplicate. Source data provided in Source Data file.

It is worth noting that interacting D1 domains from neighbouring SLP_L_ molecules completely cover the widest cavity in the SLP_H_ CWB2s tiling. This interface, defined by neighbouring CWB2_1_-CWB2_2_ motifs, at around 20 Å wide but spanning over 100 Å across the triangular prism tiles, is also hydrophilic, with complementary charges (Fig. 6a). The SLP_H_ CWB2 motif tiling also creates a cavity of ~70 Å between symmetry-related molecules which is partly occluded by the HID and LID domains, with the interlocking D1 domain ridges covering this gap (Fig. 6a). If the interacting D1 domains are flexible and can at least partially expose these cavities, it could potentially allow diffusion of larger, charged molecules through the S-layer.

Surprisingly, strain RΔD2 displays susceptibility to lysozyme, unlike the parental R20291 wild-type, which is completely resistant (Fig. 6b). Importantly, strains devoid of SlpA had been previously reported to also be lysozyme-sensitive^13^. Although lysozyme has an average diameter of about ~30 Å and the pores observed in the RΔD2 strain are only about 16 Å, absence of D2 seems to be sufficient to allow lysozyme to kill *C. difficile*. It is possible that less stable D1-LID/HID interactions with the CWB2s due to absence of the D2 domain leads to wider openings that render the S-layer permeable to lysozyme at discrete positions. In any case, our work points to an important role of D2 in preventing access of at least some antimicrobials, although the precise mechanism is still unclear.

The fitting of our H/L crystal model in the in situ EM reconstruction shows that a similarly tight packing is present in the cells. It is important to note that, unlike a previously reported electron microscopy analysis of S-layer, which included nanobodies intercalated among SbsB molecules^2^, our EM reconstruction is calculated from the native S-layer, containing not only H/L complexes but possibly other CWPs as well. However, as H/L constitutes 80–95% of the S-layer protein content, the contributions of the other CWPs are likely to have averaged out in the final reconstruction. Moreover, our reconstructions have been calculated from relatively small, well-ordered regions of the S-layer; it is possible that these regions are enriched in H/L. Any differences in packing and pore size would also not necessarily be visible after averaging.

### S-layer assembly: how can a 2D crystal array bend around a cell?

To act efficiently as a molecular sieve, the paracrystalline S-layer must be accommodated around the curved surface of the *C. difficile* cell and allow cell growth and division. Our recent work showed formation of *C. difficile* S-layer at specific sites that coincide with cell wall synthesis^26^, suggesting discrete S-layer assembly points. Indeed, dynamic flexibility between S-layer protein domains has been shown to promote efficient crystal nucleation on the curved cellular surface in *Caulobacter crescentus*^27^. Fourier analysis of S-layer ghosts, and tomographic imaging (Supplementary Fig. 9), indicate a highly mosaic surface, with many crystal defects, particularly at the cell poles, where the paracrystalline array must be distorted to allow for cell curvature. The observed pattern of crystalline patches with grain boundaries is consistent with the random secretion of S-layer protein monomers and self-assembly of 2D crystals occurring at gaps and grain boundaries within the curved S-layer, as proposed for other organisms^28,29^. It is possible that these mismatch points are wider than the observed pores and would therefore be sufficient for access of proteins and other molecules. However, whether this is the only mechanism for import/export of molecules across the S-layer and how these processes can be controlled are key questions to pursue to further our understanding of S-layer in *C. difficile*.

## Discussion

Here we report the experimentally determined structure of a complete S-layer from a medically significant pathogen, which allows us to understand the organisation of the paracrystalline array at an atomic level. The packing of H/L complexes in the crystal replicates the assembly of the functional S-layer observed in situ by electron microscopy.

One of the most surprising features of the S-layer in *C. difficile* is its compactness. While other S-layer assemblies proposed to date^2,5,6^ suggest arrays porous enough (30–100 Å) to allow substantial molecules to be imported or exported, pores in the *C. difficile* S-layer would only allow passage of small metabolites or hydrated ions. While the assembly domain SLP_H_ tiles create a 2D array, the SLP_L_ ridges cover the pores present within the triangular SLP_H_ prism packing, generating a structure impermeable to large molecules, including folded proteins such as lysozyme. How large molecules, such as the *C. difficile* toxins^8^ and other secreted proteins, are exported to the environment and how nutrients are acquired remains unclear. Moreover, S-layers must also be able to accommodate cell growth and division, while adapting to the curvature of the cellular poles. Having tightly packed core subunits or domains, maintained by interchangeable electrostatic interactions, decorated with more flexible regions, is a simple and effective way to achieve both requirements. Points of mismatched symmetry, as observed by tomography (Supplementary Fig. 9), could provide increased flexibility and permeability, perhaps creating discrete points for import of nutrients and export of larger molecules.

Currently known S-layer structures show great variability in terms of domain organisation, fold and assembly, as well as little sequence conservation across species^30,31^. Surprisingly, the tight packing of SlpA goes against our understanding of the need for a degree of permeability of the molecular sieve. Nevertheless, comparing the different known structures of individual S-layer domains and proposed assemblies does reveal some common features. Notably, all known anchoring domains, from *B. anthracis*^19^ and *Paenibacillus alvei*^32^ SLHs to the *Geobacillus stearothermophilus* secondary cell wall polysaccharide binding motif^18^, and *Caulobacter crescentus* C-terminal domain^6^, have an α-helical fold. Conversely, known assembly domains rely on β-strand rich protomers that pack to create an array, with some domains extending to link neighbouring molecules^2,3,5,18,33^. Strikingly, in *C. difficile*, the assembly and anchoring functions are combined in the largely α-helical SLP_H_ but the β-strand-rich, environment-exposed, SLP_L_ also contributes to assembly contacts. Therefore, the preference for helical domains facing the cell wall and β-strand rich external domains seems to be maintained. Moreover, a two-level organisation of domains creating a 2-layer array, was reported recently using cryo-electron tomography and cryo-EM imaging of whole cells of *B. anthracis*, *Sulfolobus* spp. and *C. crescentus*^5,6,25^. This feature is also seen in *C. difficile* (Fig. 3b), further suggesting that these might be general S-layer features. Further structural and functional studies of other S-layers will improve our understanding of the role and mechanisms of these fascinating 2D arrays.

Importantly, our characterisation of the S-layer assembly in *C. difficile* also reveals new potential therapeutic avenues. Recently reported antibodies targeting a conserved region of SLP_H_^34^ recognise a region in CWB2_3_ facing the cell wall. It will be interesting to investigate if these antibodies might disrupt the S-layer in *C. difficile*, as has been observed in *B. anthracis* using nanobodies^5^. If that is the case, molecules which affect S-layer assembly, by targeting the interacting SLP_H_ subunits and the flexible D2 domains, are attractive therapeutic agents.

## Methods

### Strains and growth conditions

*C. difficile* and *E. coli* strains are described in Supplementary Table 4. *E. coli* strains were routinely grown at 37 °C in LB broth and on LB agar (VWR or Fisher Scientific). *C. difficile* strains were routinely grown under anaerobic conditions at 37 °C on brain heart infusion (BHI, Oxoid) or supplemented BHI (BHI-S)^35^ agar and in TY broth. Growth media were supplemented with chloramphenicol (15 μg ml^−1^), thiamphenicol (15 μg ml^−1^) or kanamycin (50 μg ml^−1^) as required.

### Construction of RΔD2

DNA oligonucleotides are described in Supplementary Table 5. Plasmid pRPF233, containing a copy of the complete *slpA* gene from *C. difficile* strain R20291 was modified by inverse PCR using oligonucleotides RF102 and RF103 to delete the coding sequence of SlpA residues 115–259 and replace with GGA GGT, encoding two glycine residues. The resulting plasmid, pOB001, was transferred to the *C. difficile* S-layer mutant strain FM2.5^13^ by conjugation^36^. FM2.5 displays an aberrant colony morphology that is easily distinguished from wild-type *C. difficile*. Recombination between the plasmid-borne *slpA* gene and the mutated copy on the chromosome was detected by reversion to normal colony morphology. Plasmid curing was confirmed by loss of thiamphenicol resistance, the chromosomal location of the engineered *slpA* gene was confirmed by PCR and the resulting protein profile was determined by SDS PAGE of S-layer proteins isolated using standard methods (see below).

### Plasmid construction

For crystallisation studies, fragments of R7404 *slpA*, encoding mature HID (residues 1–41; nucleotides 1024–1065) and SLP_L_ (residues 1–316; nucleotides 73–1023) or LID (residues 240–316; nucleotides 793–1023) were amplified from genomic DNA and cloned into pACYC-Duet1 yielding plasmids pJAK149 and pJAK147, respectively. C-terminally 6xHis-tagged HID was amplified using RF1396 and RF1397 and cloned into pACYC-Duet (MCS1) linearised using RF1398 and RF1400 using Gibson assembly, and SLP_L_ or LID were amplified using RF1394 and RF1395 or RF1395 and RF1396, respectively, and cloned into MCS2 using *Nde*I-*Kpn*I restriction cloning.

To study protein-protein interactions in vitro, DNA encoding mature SLP_L_ or SLP_H_ of R7404 was amplified using Q5 (NEB) PCR and cloned into pET28a using *Nco*I-*Xho*I restriction cloning, in frame with a C-terminal 6xHis-tag. Deletion variants lacking HID or LID or point mutants within HID and LID were constructed by inverse PCR, using primers listed in Supplementary Table 5. To study the impact of individual LID and HID point mutations on H/L complex assembly in *C. difficile*, codons for SLP_L_ F274 or SLP_H_ Y27 in pRPF170 were mutated to GCA (Ala) by inverse PCR cloning, yielding plasmids pRPF209 and pJAK186 respectively.

### Protein expression and purification

S-layer was extracted as previously described^15^. Briefly, 400 ml of *C. difficile* CD630, R7404, RΔD2 16-h culture were harvested by centrifugation at 4696 × *g* for 30 min at room temperature. Cells were washed with 20 ml of phosphate-buffered saline (PBS) pH 7.4, centrifuged for 10 min at 4696 × *g*, and resuspended in 5 ml of 0.2 M glycine-HCl pH 2.2. Cell suspension was centrifuged at 21,100 × *g* for 10 min, and recovered supernatant was neutralised with 2 M Tris-base. S-layer extract was then filtered and resolved onto a Superdex 200 26/600 column using an ÄKTA Pure FPLC system (GE Healthcare) in 10 mM Tris-HCl pH 7.5, 100 mM NaCl, 5 mM EGTA buffer.

BL21 (DE3) cells transformed with plasmids pJAK149 or pJAK147 were used to co-express HID-6xHis-tag and SLP_L_ or HID-6xHis-tag and LID, in Auto Induction Media TB (Formedium) supplemented with 30 µg ml^−1^ chloramphenicol. Cells from 1 L of culture grown for 18 h at 37 °C were harvested by centrifugation at 4000 × *g*, 4 °C for 30 min. Pellets were washed with PBS and frozen in −20 °C.

Variants of SlpA subunits (Supplementary Table 4) for interaction studies were expressed in Rosetta (DE3) cells, in 50 ml of Merck Novagen Overnight Express Instant TB Medium, supplemented with 50 μg ml^−1^ kanamycin and 15 μg ml^−1^ chloramphenicol for 18 h at 37 °C. Harvested cells were washed with PBS and frozen at −20 °C.

Cell lysis was performed using BugBuster Protein Extraction Reagent (Novagen). Pellets were resuspended in lysis buffer (50 mM Tris-HCl pH 8.0, 250 mM NaCl, 1x cOmplete EDTA-free protease inhibitors (Roche), 100 μg ml^−1^ lysozyme, 10 μg ml^−1^ DNase I, 1x BugBuster) and incubated at room temperature for 30 min. Extracts were centrifuged at 20,000 × *g* for 30 min, supernatant was filtered and separated on a 5 ml HisTrap (GE Healthcare) column in 50 mM Tris-HCl pH 8.0, 250 mM NaCl, 10 mM imidazole, with a linear gradient of imidazole (10–500 mM). Eluate fractions were further purified using size exclusion chromatography on Superdex 200 (GE Healthcare) in 50 mM Tris-HCl pH 7.5, 150 mM NaCl.

Variants of SLP_H_ were recovered by affinity chromatography from inclusion bodies, solubilized in 50 mM Tris-HCl pH 7.5, 500 mM NaCl and 8 M urea for 20 min at room temperature. Solubilized, cleared supernatant was loaded onto 5 ml HisTrap (GE Healthcare) column in solubilisation buffer, and column-bound protein was refolded in 50 ml of 50 mM Tris-HCl pH 7.5, 500 mM NaCl, 0.1% Triton X-100, followed by 50 ml of 50 mM Tris-HCl pH 7.5, 250 mM NaCl, 5 mM β-cyclodextrin. Affinity purification was performed in 50 mM Tris pH 8.0, 250 mM NaCl, 10 mM imidazole, with protein elution by a linear gradient of imidazole (10–500 mM).

### Protein analysis by western immunoblotting

For analysis of H/L complex interactions on the surface of *C. difficile*, plasmids carrying a tetracycline-inducible copy of CD630 *slpA* (pRPF170) or derivatives with a point mutation in the LID (F274A, pRPF209) or the HID (Y27A, pJAK186) were transferred into the *slpA* null strain FM2.5 by conjugation^36^. Strains were grown to an OD_600 nm_ of ~0.4 in TY broth and induced with anhydrotetracycline (20 ng ml^−1^). Surface localised H/L subunits were extracted using low pH glycine as described above and normalised to an equivalent OD_600 nm_ of 25. Culture supernatants were filtered, concentrated to an equivalent OD_600 nm_ of 50 using a Vivaspin column with a 10 kDa MWCO. Samples were then subjected to SDS PAGE and western immunoblotting was carried out using polyclonal rabbit antibodies specific for the CD630 SLP_H_ (dilution 1:200,000) or SLP_L_ (dilution 1:100,000)^15^ and detected using goat HRP-conjugated secondary anti-rabbit antibody (Invitrogen) used at 1:10,000.

### Analysis of protein-protein interactions by enzyme-linked immunosorbent assay

The assays were performed as previously described^15^. Briefly, Maxisorp microtiter plates (Nunc) were coated with 10 μg ml^−1^ of SLP_L_ or SLP_H_ and their variants (Supplementary Table 4), blocked with 3% (w/v) milk in PBS/ 0.05%Tween-20, and overlaid with respective interacting partner SLP_L_ or SLP_H_ across 0.0001–100 μg ml^−1^ range. Binding was assessed with polyclonal rabbit primary antibodies against the overlay protein (α-SLP_L_ at 1:30,000 and α-SLP_H_ at 1:15,000 dilution). Spectrophotometric detection of product of horse radish peroxidase (HRP-conjugated secondary anti-rabbit antibody used at 1:2500 dilution) with *o-*phenylenediamine dihydrochloride (OPD; 1 mg ml^−1^ in solution of 20 mM citric acid, 50 mM Na_2_HPO_4_) upon addition of 0.04% (v/v) hydrogen peroxide to the reaction mix was carried out at 490 nm using Biotek ELx800 plate reader.

### X-ray crystallography

Purified and concentrated proteins (recombinant LID/HID-6xHis-tag at 38 mg ml^−1^ and SLP_L_/HID-6xHis-tag at 21 mg ml^−1^, CD630, R7404 and RΔD2 H/L at 10 mg ml^−1^) were subjected to crystallisation using a Mosquito liquid handling robot (TTP Labtech), with the sitting drop vapour-diffusion method at 20 °C. Native H/L complex crystallised in 0.1 MES pH 6, 1.25 M lithium chloride, 16 PEG 6000 and 10 % glycerol. Recombinant SLP_L_/HID-6xHis-tag produced diffraction quality crystals in 0.2 M ammonium sulphate, 0.1 M MES pH 6.5, 35 % MPD, while LID/HID-6xHis-tag was crystallised in 1.6 M sodium citrate tribasic dihydrate pH 6.5. Data were collected on the I04 (*λ* = 0.98 Å), I23 (*λ* = 2.75 Å) and I24 (*λ* = 0.97 Å) beamlines at the Diamond Light Source Synchrotron (Didcot, UK) at 100 K. The data were acquired from the automatic software pipeline xia2 within the Information System for Protein Crystallography Beamline (ISPyB), processed with XDS^37^, iMosflm^38^ or DIALS^39^ and scaled with Aimless^40^ within CCP4i^41^ or CCP4i2^42^ software suits. When needed, density modification was performed with PARROT^43^.

The structure of LID/HID was solved de novo using Arcimboldo_lite^44^ within CCP4i, starting from several 10–14 residues-long polyalanine models of α-helices. Automatic model building was performed with Buccaneer^45^, followed by manual building with Coot^46^ and refinement with Phenix_refine^47^.

The structure of SLP_L_/HID-6xHis-tag was determined by sequential molecular replacement in Phaser^48^ searching first for SLP_L_ D1-D2 domains model (3CVZ^15^), followed by the search with LID/HID structure into a fixed SLP_L_ solution, and subsequent manual building (COOT) and refinement (Phenix_refine).

Initial attempts to solve the substructure of the complete H/L complex by S-SAD provided only weak phases, and were improved by combining molecular replacement in Phaser using the CWB2 domain core of *C. difficile* Cwp8 (PDB ID: 5J6Q^17^) with sulphur anomalous difference Fourier maps using Anode^49^. This solution was used for MR-SSAD in Phenix.autosol and cycles of manual building in COOT and density improvement were used to improve the electron density maps and the model of the core SLP_H_. A complete H/L model was obtained by successive molecular replacement runs using Phaser in CCP4i2 combining the SLP_H_ model with the obtained LID/HID, D2 from SLP_L_/HID structure and D1 from CD630 SLP_L_ (PDB ID: 3CVZ) with loops removed. Final models were obtained after iterative cycles of manual model building with Coot and refinement in Phenix_refine and REFMAC5^50^ as well as PDB-REDO^51^. Applied strategies included refinement of XYZ coordinates, real space, individual B-factors, TLS parameters and occupancies. Validation of final models was performed using COOT and Phenix internal tools, as well as MOLPROBITY^52^ web server. Data collection and refinement statistics are summarised in Supplementary Table 1. Structural representations were generated using PyMOL Molecular Graphics System (Schrödinger, LLC) or Chimera 1.13.1^53^.

### Electron crystallography data collection

To allow visualisation by electron microscopy, S-layers were either removed from *C. difficile* cells in a single piece following peptidoglycan digestion (S-layer ghosts) or cells were mechanically fragmented (S-layer/cell wall fragments). *C. difficile* cells were harvested by centrifugation and resuspended to an OD_600nm_ of 10 in 20 mM HEPES pH 7.5, 150 mM NaCl, 500 mM sucrose. For S-layer ghosts, cell walls were digested using purified ϕCD27 endolysin for 30 min at 37 °C^54^. The resulting membrane-bound spheroplasts were removed from the sample by centrifugation at 2000 × *g* for 2 min and the supernatant, containing S-layer ghosts, was retained for imaging. 5 μl of S-layer ghosts were loaded on glow-discharged, amorphous carbon-coated 300 mesh copper EM grids and stained with 2% uranyl acetate, as previously described^55^. Samples were examined on a Phillips CM200 FEG transmission electron microscope at 200 kV. Images were collected on a 4096 × 4096 pixel Gatan UltraScan 4000SP Model 890 CCD camera (Gatan Inc.), with 15 µm pixel size. A total of 36 micrographs of R20291 and 29 micrographs for RΔD2 S-layer extracts were collected at a magnification of ×82,351 and defocus range from −800 to −2200 nm. The specimen tilt angle ranged from −55° to +55° in increments of 10°.

For cryo-EM, fragments of S-layers were generated by mechanical disruption. Briefly, 60 ml of *C. difficile* cells at OD_600nm_ 0.6–0.8 were centrifuged at 2000 × *g* for 15 min at 4 °C. The cell pellet was washed twice in ice cold deionised water and combined with an equal volume of pre-cooled acid-washed glass beads (Sigma) and homogenised in a Braun MSK homogenizer for 30 s. The homogenate was cooled and centrifuged at 800 × *g* for 10 min to remove glass beads and unbroken cells. S-layer fragments were then harvested at 3000 × *g* for 10 min, washed with cold 1 M NaCl and resuspended in cold 2% Triton X-100. 2.5 µl of the S-layer fragments were added to glow-discharged Quantifoil® 2/2 grids. The grids were then blotted for 30 s and plunged into liquid ethane, using a Vitrobot Mark III (FEI). The frozen grids were stored in liquid nitrogen for later observation. Micrographs, at ×68,000 magnification and defocus range of −2000 to −3000 nm, were obtained on a Falcon II direct electron detector (FEI) using a Tecnai F20 microscope (FEI) operating at 200 keV. For whole-cell imaging, 1 ml overnight culture was centrifuged at 4000 × *g*, the pellet was washed twice in PBS buffer, and then resuspended to an OD_600nm_ of 10. After application of 3 μl of the sample to a glow-discharged Quantifoil R2/2 grid, the cell was imaged on a Technai Arctica 200 kV microscope.

### Electron crystallography data processing

Images were initially processed using the *2dx* suite^56–58^. Most micrographs of S-layer ghosts showed two rotationally separated lattices in Fourier transforms and these were indexed independently. Images were masked based on crystal size and good crystalline order and subjected to two cycles of unbending using the programmes *QUADSEARCH* and *CCUNBEND*. The symmetry was determined from images of untilted crystals using *ALLSPACE*^59^. Phase origins for individual images were refined against each other using *ORIGTILTK*, sequentially adding images of higher tilt to the refinement. Crystal tilt angles were estimated from lattice distortion. *LATLINE*^60^ was used to determine interpolated amplitudes and phases on a regular lattice of 1/160 Å^−1^ in the z* direction. A Gaussian tapered real‐space envelope of width slightly larger than that of the SlpA molecule estimated from the X-ray crystal structure (70 Å for wild type and 60 Å for RΔD2) was applied. The phase origin and tilt parameters were further refined using the output interpolated lattice lines as reference. The variation of amplitude and phase along *0*,*0*,*l* was estimated by examining a plot of maximum contrast on each Z-section in real space^61^. The final structure factors were sampled from the interpolated lattice lines^60^ and a 3D map generated within the CCP4 suite of programmes^41,57^. Cryo-EM micrographs of untilted R20291 and RΔD2 samples were processed similarly to generate 2D projection maps. B-factors were calculated using *SCALIMAMP3D* with bacteriorhodopsin diffraction amplitudes as reference^62^. Final maps are available in EMDB repository under access code EMD-13957.

### Fitting X-ray structures to EM density

The SlpA_R20291_ and SlpA_RΔD2_ X-ray structure co-ordinates were fitted using Chimera 1.13.1^53^ into the respective electron microscopy reconstructions. The extended lattice of each SlpA was generated by calculating symmetry-related molecules from the crystal packing in Pymol, and then manually oriented in the EM density based on the known surface orientation *i.e*. SLP_L_ facing the environment, and SLP_H_ facing the cell wall. The *Fit in map* function was then used to calculate the highest correlation to a map simulated from the X-ray structure co-ordinates at 20 Å resolution. To illustrate that the overall EM envelope could represent a small movement in the SLP_L_ domains, further modelling was performed using ISOLDE^63^ to flexibly fit the previous lattice model into the negative stain map using torsion and distance restraints, a temperature of 100 K and reducing the map weight to 0.02915. Restraints allowed movement of the domains whilst maintaining overall domain structure, determined by the RMSD between original and fitted domains (SLP_H_ RMSD 1.07 Å, LID/HID RMSD 0.69 Å, SLP_L_ D1 domain RMSD 0.58 Å, and SLP_L_ D2 domain RMSD 1.16 Å; final fitted model PDB ID: 7QGQ).

### Tomography

For cryo-electron tomography (cryo-ET), the homogenised S-layer ghost sample used in electron crystallography was mixed with an equal volume of 10 nm BSA-treated nanogold beads, and 3 μl of this mixture was applied to a glow-discharged lacey carbon with ultra-thin carbon 300 mesh grid, blotted for 3 s and plunged into liquid ethane, using a Leica EM GP. The frozen grids were stored in liquid nitrogen temperature for later observation. Tilt series were collected on a Titan Krios microscope operating at 300 keV with a GIF Quantum energy filter, Volta phase plate, and K2 camera operating in super-resolution mode. Micrographs were collected using SerialEM, at a pixel size of 5.47 Å, with each tilt series covering ±60° with a tilt increment of 3° and collected with a grouped dose-symmetric acquisition scheme and group sizes of 4. Samples from each tilt series received 100 e/Å^2^ total dose with 20 frames per tilt. Tomograms were constructed using the IMOD package^64^. Tilt series were tracked and aligned based on fiducial markers, and then tomograms were reconstructed by weighted back projection with 1x binning.

### Lysozyme resistance

To assess resistance to lysozyme, overnight *C. difficile* cultures were grown in TY broth, subcultured to an OD_600nm_ of 0.05 in 1 ml fresh TY in a 1.5 ml cuvette and then grown for 8 h with hourly OD_600nm_ measurements. Where appropriate, lysozyme (500 μg ml^−1^) was added after 2.5 h growth. Experiments were performed in triplicate on biological duplicates and data expressed as the mean and standard deviation.

### Other methods

Sequences of SLCTs were downloaded from *C. difficile* Multi Locus Sequence Typing website (https://pubmlst.org/cdifficile/) and aligned using MAFFT. Multiple sequence alignments were visualised and annotated using Jalview or ALINE^65^. Analysis of the evolutionary conservation of amino acids was performed using ConSurf webserver (https://consurf.tau.ac.il/) and visualised onto crystal structure of CD630 H/L complex (PDB ID 7ACY) to map the conservation scores onto 3D crystal structure using PyMOL. Conservation scores were calculated with the Bayesian method, using the WAG model of amino acid substitution (selected based on ProtTest 3.4.1). PDBeFold^66^ was used to compare the similarity between SlpA models determined in this study. PISA^67^, PDBSum^68^ and LigPlot^+ ^^69^ were used to investigate interdomain and protein-protein interfaces. Structural flexibility of models was assessed by DynDom6D (v1.0 with default settings^70^), HingeProt webserver^71^ and the components of DynOmics webserver. Homology models for SLCT representatives^13^ were generated by providing SWISS-MODEL webserver with the H/L complex crystal model as a user template. Structural alignments between homology model and template were performed using COOT. Data was analysed using Numpy v1.16.6^72^, Pandas v0.24.2^73^ and heatmap generated with Seaborn v0.10.1^74^.

Analysis of pores in the H/L array was carried out using ChexVis^75^ and hydrophobicity patterns for residues lining each pore calculated using the Kyte-Doolittle scale^76^.

### Reporting summary

Further information on research design is available in the Nature Research Reporting Summary linked to this article.

## Supplementary information

Supplementary Information

Description of Additional Supplementary Files

Supplementary Movie 1

Reporting summary

## Data Availability

X-ray structural data are available in the PDB repository under PDB IDs: 7ACW, 7ACV, 7ACX, 7ACY and 7ACZ. Electron crystallography data are available in the EMDB repository under accession code EMD-13957 and the fitted model is available in the PDB repository with PDB ID: 7QGQ. Source data are provided with this paper. Any other datasets generated and/or analysed during the current study are available from the corresponding authors on request. Source data are provided with this paper.

## Source data

Source Data
