# Supplementary material for: Structure and assembly of the S-layer in C. difficile
Source: Nat Commun. 2022 Feb 25;13:970. doi: 10.1038/s41467-022-28196-w (PMC8881574; doi:10.1038/s41467-022-28196-w)
Supplement: Supplementary file 1 — Supplementary Information [file 41467_2022_28196_MOESM1_ESM.pdf]

SUPPLEMENTARY INFORMATION

Structure and assembly of the S-layer in *C. difficile*

Paola Lanzoni-Mangutchi[1]*, Oishik Banerji[2*#], Jason Wilson[2]*, Anna Barwinska-Sendra[1]*, Joseph A. Kirk[2,3], Filipa Vaz[4‡], Shauna O'Beirne[2,3], Arnaud Baslé[1], Kamel El Omari[5], Armin Wagner[5], Neil F. Fairweather[6], Gillian R. Douce[4], Per A. Bullough[2§], Robert P. Fagan[2,3§], Paula S. Salgado[1§]

[1] Biosciences Institute, Faculty of Medical Sciences, Newcastle University, Newcastle upon Tyne, UK

[2] Krebs Institute, School of Biosciences, University of Sheffield, Sheffield, UK

[3] Florey Institute, School of Biosciences, University of Sheffield, Sheffield, UK

[4] Institute of Infection, Immunity and Inflammation, College of Medical, Veterinary and Life Sciences, University of Glasgow, Glasgow, UK

[5] Diamond Light Source, Oxfordshire, UK

[6] Dept. Of Life Sciences, Imperial College London, London, UK

[#] Current address: Royal Society of Chemistry, Burlington House, Piccadilly, London, UK

[‡] Current address: Department of Immunology, Oslo University Hospital, Oslo, Norway

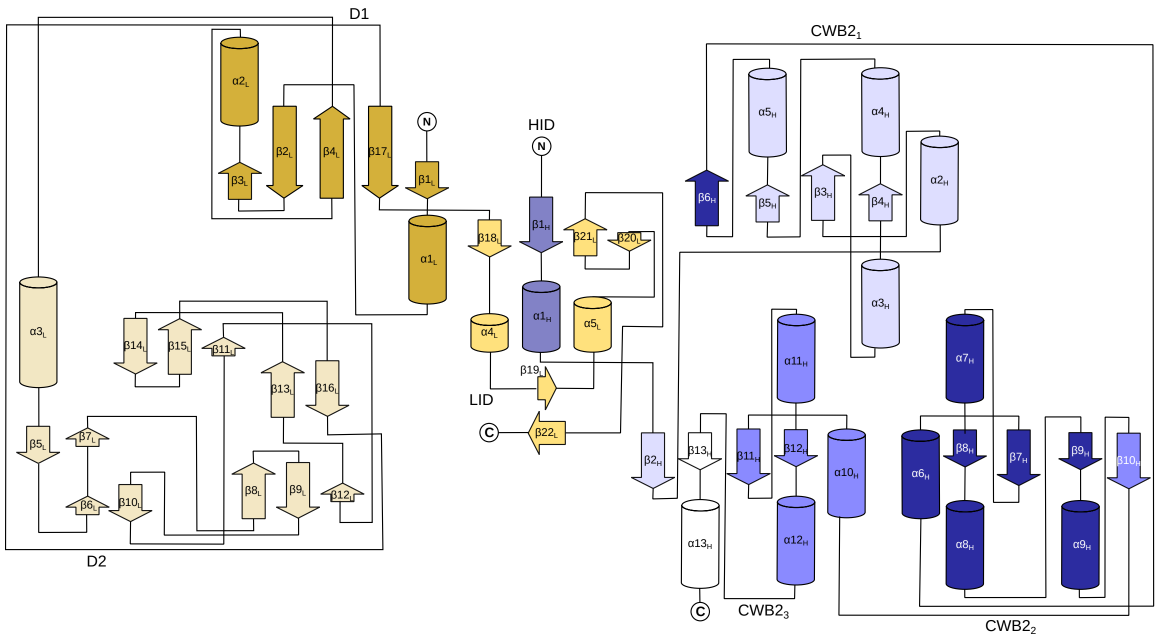

Supplementary Fig. 1: H/L complex topology

Topology of the mature $SlpA_{CD630}$ H/L complex, retrieved from structure analysis with PBDsum and Coot. SLP_L represented in gold and SLP_H coloured in blue. Colour shading represents different domains/motifs. Numbering of secondary structure components based on PDB ID 7ACY, subscripts indicate the relevant subunit. D1 – SLP_L domain 1, D2 – SLP_L domain 2, LID – SLP_L interacting domain, HID – SLP_H interacting domain, $CWB2_1$ – cell wall binding domain 1, $CWB2_2$ – cell wall binding domain 2, $CWB2_3$ – cell wall binding domain 3.

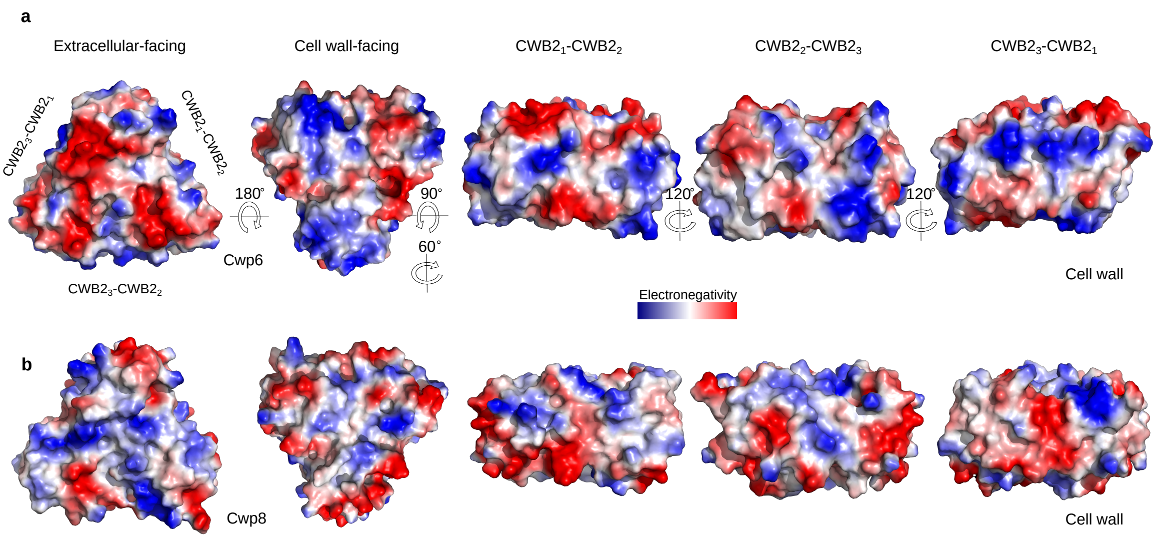

Supplementary Fig. 2: Charge distribution across CWB2 motifs in CWP6 and CWP8, two minor components of the *C. difficile* S-layer

Comparison of the Poisson-Boltzmann electrostatic potential calculated for CWB2 motifs from Cwp6 (a) and Cwp8 (b). The triangular CWB2 motifs of each CWP were superimposed onto the $SlpA_{CD630}$ CWB2s to determine orientation of Cwp6 and Cwp8. Views are shown from the extracellular and cell wall surfaces, followed by side views of the lateral faces defined by two interacting CWB2s, as per $SlpA_{CD630}$ H/L complex orientation at the cell surface. Electronegativity gradient: positive in blue to negative in red.

a

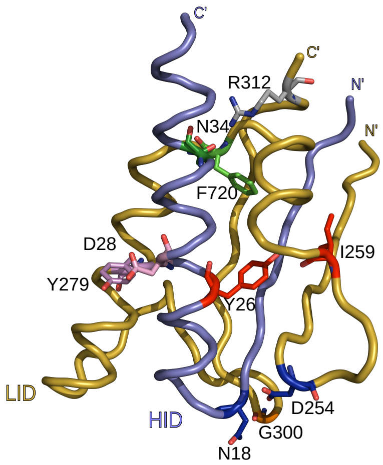

SLP_L			SLP_H	
Residue	Atom	Distance, Å	Residue	Atom
Asp254	$O\delta^2$	2.75	Asn18	N
Gly300	O	3.07	Asn18	$N\delta^2$
Ile259	O	2.73	Tyr26	$O\eta$
Phe270	O	2.96	Asn34	$N\delta^2$
Tyr279	$O\eta$	3.82	Asp28	$O\delta^2$
Phe270	N	3.07	Asn34	$O\delta^1$
Arg312	$N\eta^2$	2.86	Asn34	$O\delta^1$

b

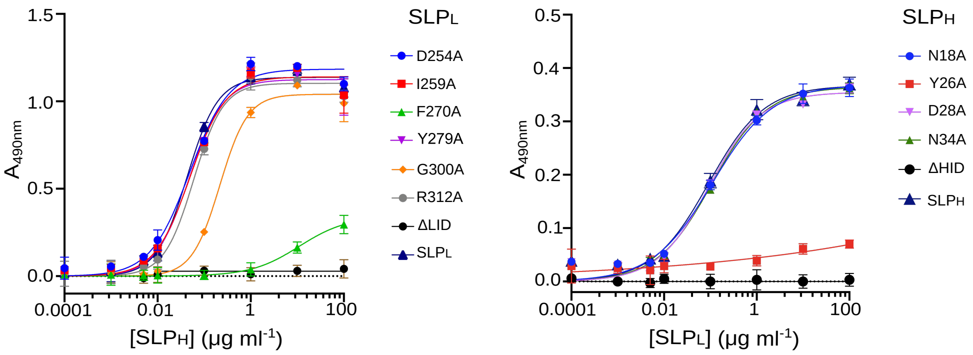

Supplementary Fig. 3: Residues required for H/L complex

a, Key interactions identified at the interface of the LID/HID complex from strain R7404 (SLCT-7b) (left; identified with PDBePISA in PDB ID: 7ACW) informed site directed mutagenesis for functional assessment.

b, Effects of point substitution mutations in SLP_L (left) or SLP_H (right) on complex formation were tested by ELISA. Graphs represent mean ± SD of n = 3 experiments, with least squares curve fit. Source data provided in Source Data file.

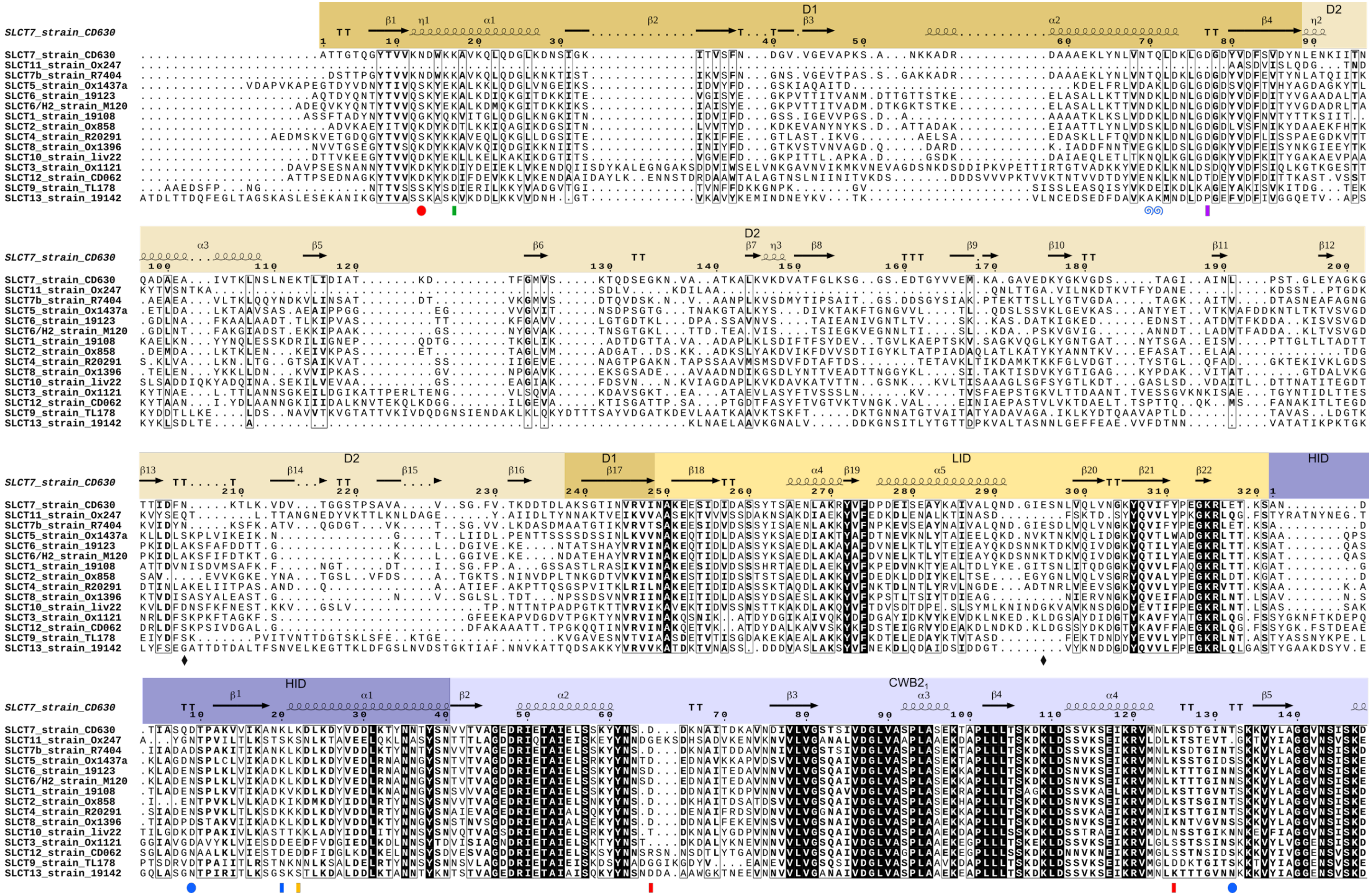
D1
D2
LID
HID
CWB2
SLCT7_strain_CD630
SLCT11_strain_0x247
SLCT7b_strain_R7404
SLCT5_strain_0x1437a
SLCT6_strain_19123
SLCT6/H2_strain_M120
SLCT1_strain_19108
SLCT2_strain_0x858
SLCT4_strain_R20291
SLCT8_strain_0x1396
SLCT10_strain_liv22
SLCT3_strain_0x1121
SLCT12_strain_CD062
SLCT9_strain_TL178
SLCT13_strain_19142

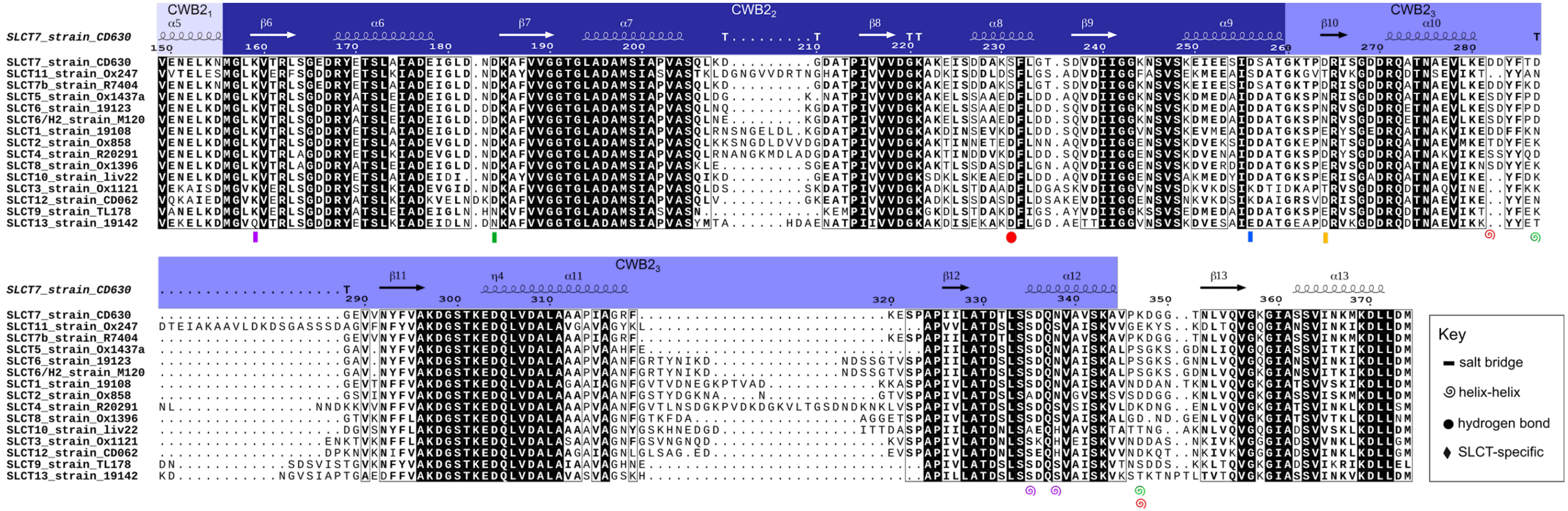

Supplementary Fig. 4: Sequence alignment of representatives of each S-layer cassette type (SLCT)

Secondary structural elements identified in the H/L CD630 complex (PDB ID: 7ACY) are indicated by looped lines – α-helices and arrows – β-sheets. SLP_L D1, D2 and LID domains and SLP_H HID domain and CWB2 motifs are highlighted, coloured as in Fig. 1. Residues involved in interactions are marked as per key (see Supplementary Fig. 5 for details), with interacting residues marked with the same colour. Strictly conserved residues across all SLCTs are highlighted in black background, partially conserved groups are delimited by a box, with residues conserved within each group highlighted in bold, as per default in ESPript3 (http://espript.ibcp.fr).

SLCT7 - strain CD630*; SLCT1 - strain 1912; SLCT2 - strain Ox858; SLCT3 - Ox1121; SLCT4 - strain R20291*; SLCT5 - Ox1437a; SLCT6 - strain 19123; SLCT6/H2 - strain M120; SLCT7b - R7404*; SLCT8 - Ox1396; SLCT9 - strain TL178; SLCT10 - strain Liv22; SLCT11 - strain Ox247; SLCT12 - strain CD062; SLCT13 - strain 19142.

*indicates strains with structural models included in this work.

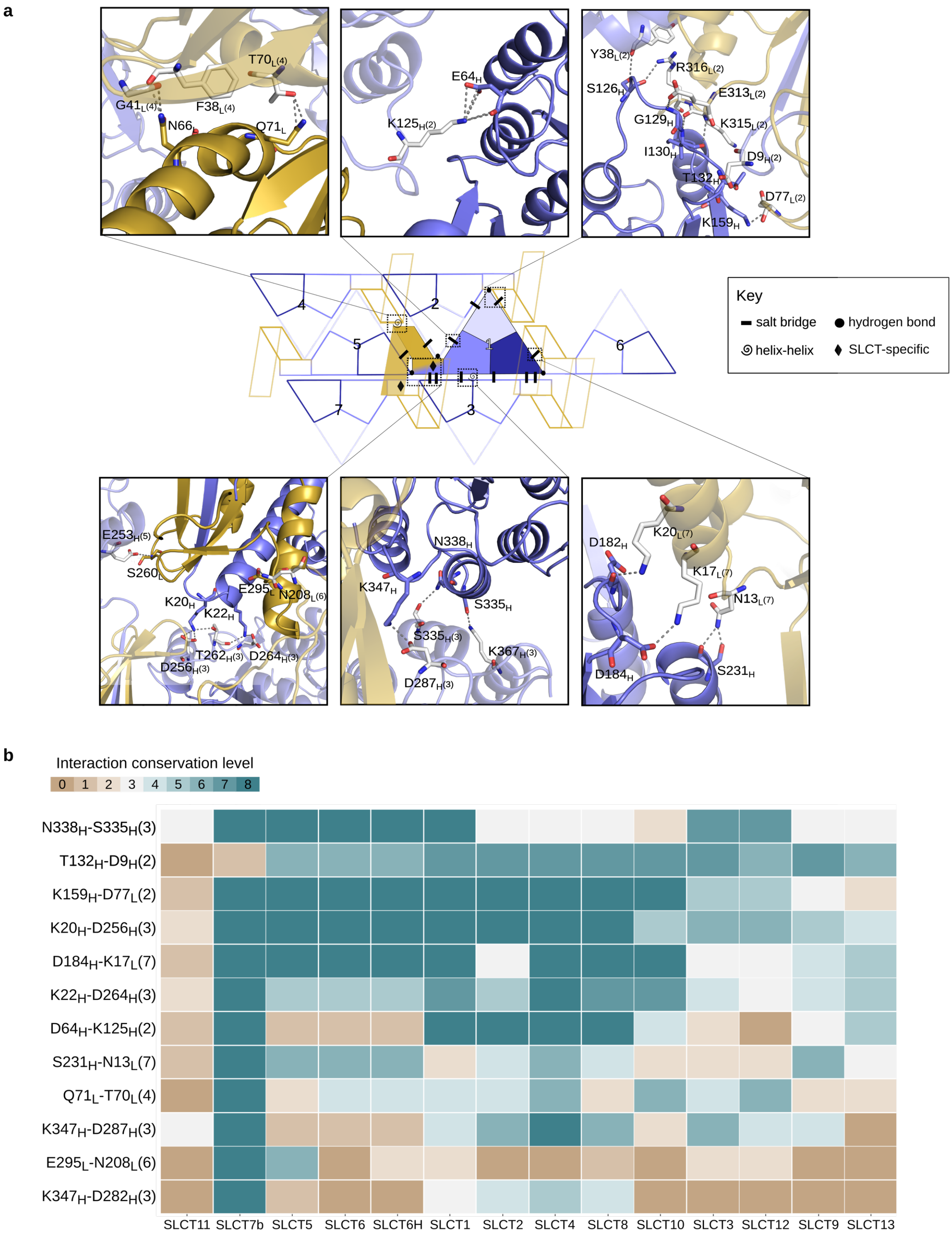

Supplementary Fig. 5: Interactions between neighbouring molecules in S-layer packing

a, Details of the interaction network between 7 SlpA$_{CD630}$ H/L complexes within the 2D array, with each molecule represented as cartoon, interacting residues as sticks (coloured as in schematic for molecule 1, white for neighbouring residues, with molecule number identifier

in parenthesis) and interactions as dashed lines. The interface depicted in each panel is marked by a corresponding box within the array representation

b, Clustermap of predicted conservation across known SLCTs for sidechain-sidechain interactions found in $SlpA_{CD630}$ H/L. Representatives of each SLCT were aligned (Supplementary Fig. 4), SWISS-MODEL structural homology models were generated and superimposed. The residues corresponding to interactions identified in $SlpA_{CD630}$ H/L were analyzed and interaction conservation compared to SLCT-7 was depicted based on residue conservation and prediction of similar or different type of possible interaction. Key: 0 – no residue conservation, unstructured region; 1 – one conserved residue, unstructured region; 2 – no residue conservation, no interaction; 3 – one conserved residue, no predicted interaction; 4 – no residue conservation, different interaction type; 5 – one conserved residue, different interaction type; 6 – no residue conservation, same interaction type; 7 – one conserved residue, same interaction type; 8 – residues and interaction conserved.

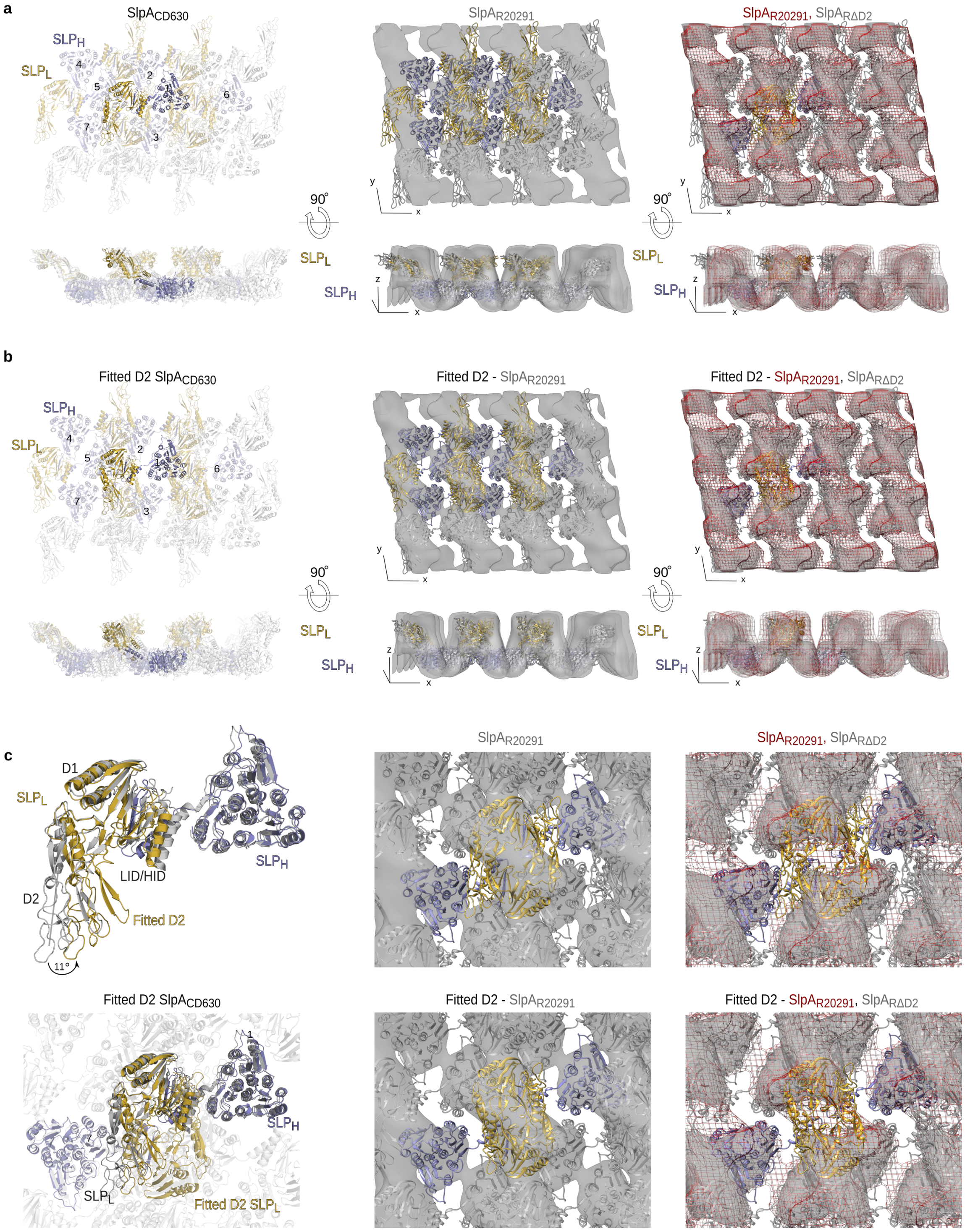

Supplementary Fig. 6: Rotation of D2 domain allows optimised fit of H/L X-ray structure into the *in situ* packing of the native S-layer

a, H/L planar crystallographic array (left, PDB ID 7ACY, cartoon representation, SLP$_L$ - gold, SLP$_H$ - slate blue, views as defined in Fig. 1b) fitting into the 3D reconstruction of negatively

stained S-layer ghost indicating the overall envelope in the native lattice (grey map, middle). Right panels show the native S-layer reconstruction as red chicken wire, overlayed on grey map for the 3D reconstruction from $SlpA_{R\Delta D2}$ ghosts after rigid body fitting of the X-ray models. Reconstructions and arrays are shown from the environment (top panels) and side views in the 2D plane (bottom panels).

b, Rotation of SLP_L domain D2 by 11° towards the D1 domain allows for an improved fitting of H/L array into the native S-layer. The overall crystal packing is maintained (left, colours and views as in **a**) and a more compact SLP_L fits the native lattice (middle panel), matching most of the density of the ridges, as seen in the side views (bottom panels). Comparison of the $SlpA_{R\Delta D2}$ (right, grey surface) and $SlpA_{R20291}$ (right, red chicken wire) reconstructions with the fitted D2 model further demonstrates that the missing density in the mutant S-layer reconstruction corresponds to the deleted D2 domain.

c, Superimposition of the crystallographic H/L complex (top left, grey) and the fitted (top left, coloured as in **a** and **b**) models shows a similar overall fold of the complex, with negligible reorientation in SLP_H, D1 and LID/HID (RMSD between original and fitted subdomains is <1Å, see Methods for details) but a significant rotation of D2 towards D1 by 11°.

Zoomed views of the structural models with original (top) and reoriented D2 domains (bottom) fitted into $SlpA_{R20291}$ (middle panels) and $SlpA_{R\Delta D2}$ (left panels) S-layer ghosts, as presented in **a** and **b** illustrate how a more compact packing is possible.

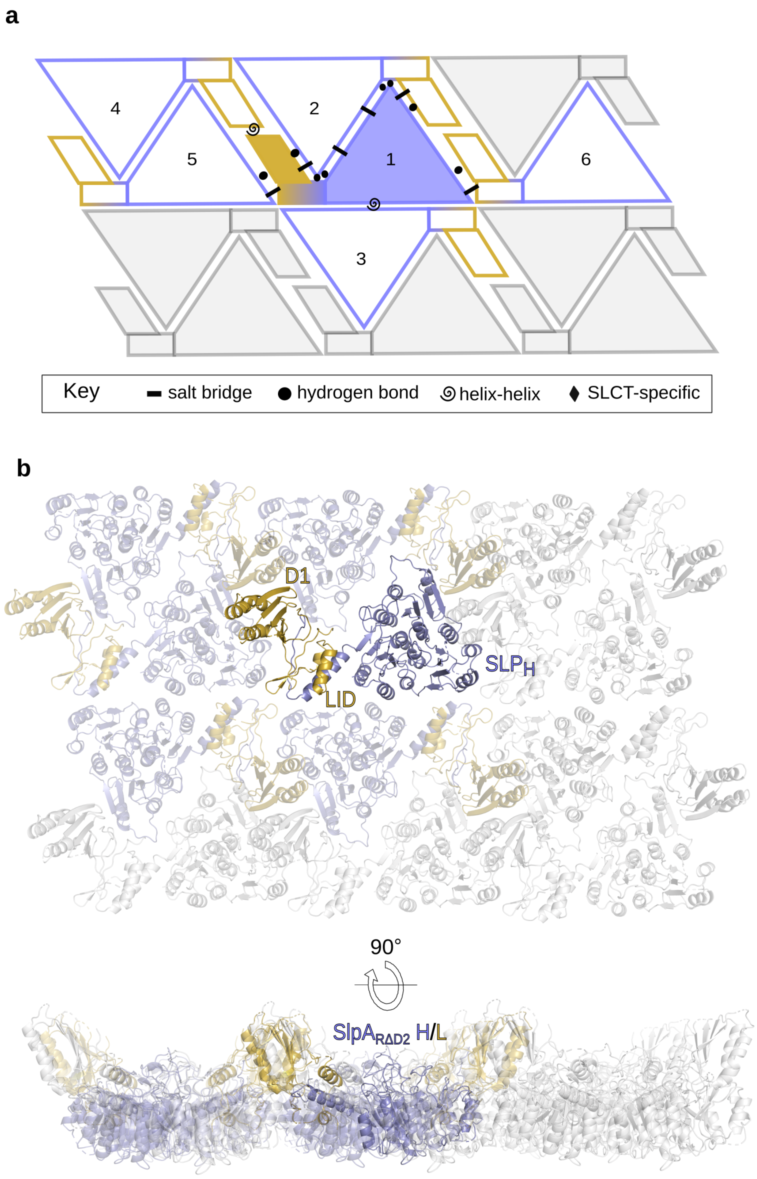

Supplementary Fig. 7: Absence of D2 does not affect overall structure and packing

a, 2D tiling representation of $SlpA_{R\Delta D2}$ assembly in crystal packing (PDB ID: 7ACZ), with identified interactions represented as symbols defined in the key (as in Fig. 3).

b, Cartoon representation of the $SlpA_{R\Delta D2}$ array (SLP_L coloured in gold and SLP_H in slate blue) in top and a side view (as defined in Fig. 3).

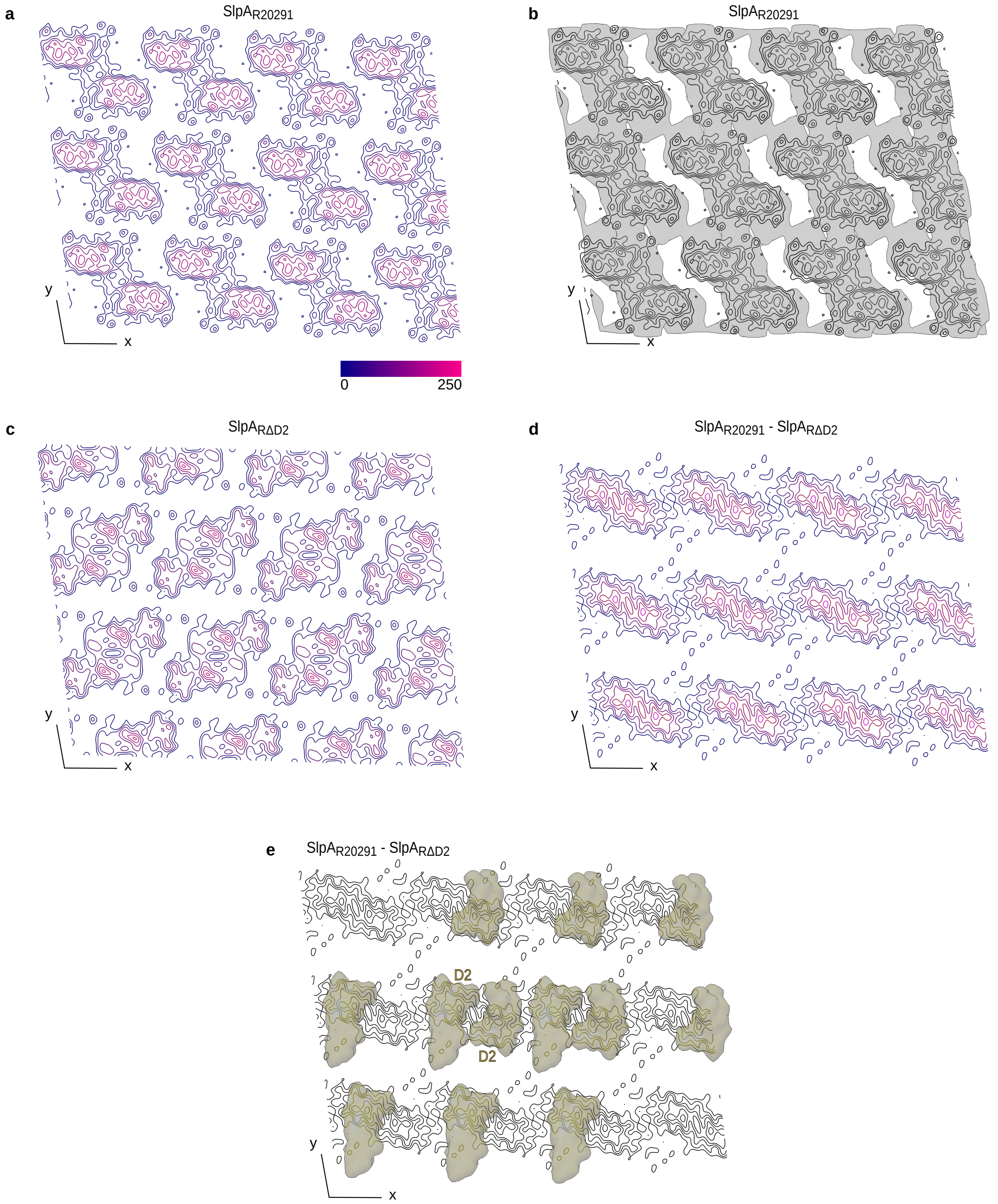

Supplementary Fig. 8: Cryo-electron microscopy of wild type and $SlpA_{R\Delta D2}$ S-layer ghosts.

a, Projection map of frozen hydrated native S-layer ghost from strain R20291 at 8.7 Å resolution. Contours represent density greater than mean density, contour interval 0.5 RMS density, as per gradient (0 – blue to pink – 250).

b, Superimposition of the reconstruction of negatively stained native S-layer (grey surface) on the projection in **a**.

c, Projection map at 8.7 Å resolution of frozen hydrated *C. difficile* S-layer ghost containing $SlpA_{R\Delta D2}$, depicted as in **a**.

d, Wild type minus $SlpA_{R\Delta D2}$ difference projection map. Positive difference density corresponds to the projection of the ridge-like density in the 3D reconstruction shown in **b**.

e, Superposition of isolated domain D2 crystal structure (gold surface) on the difference projection map in **d**. Pseudo-symmetrically related structures are shown together in the central density.

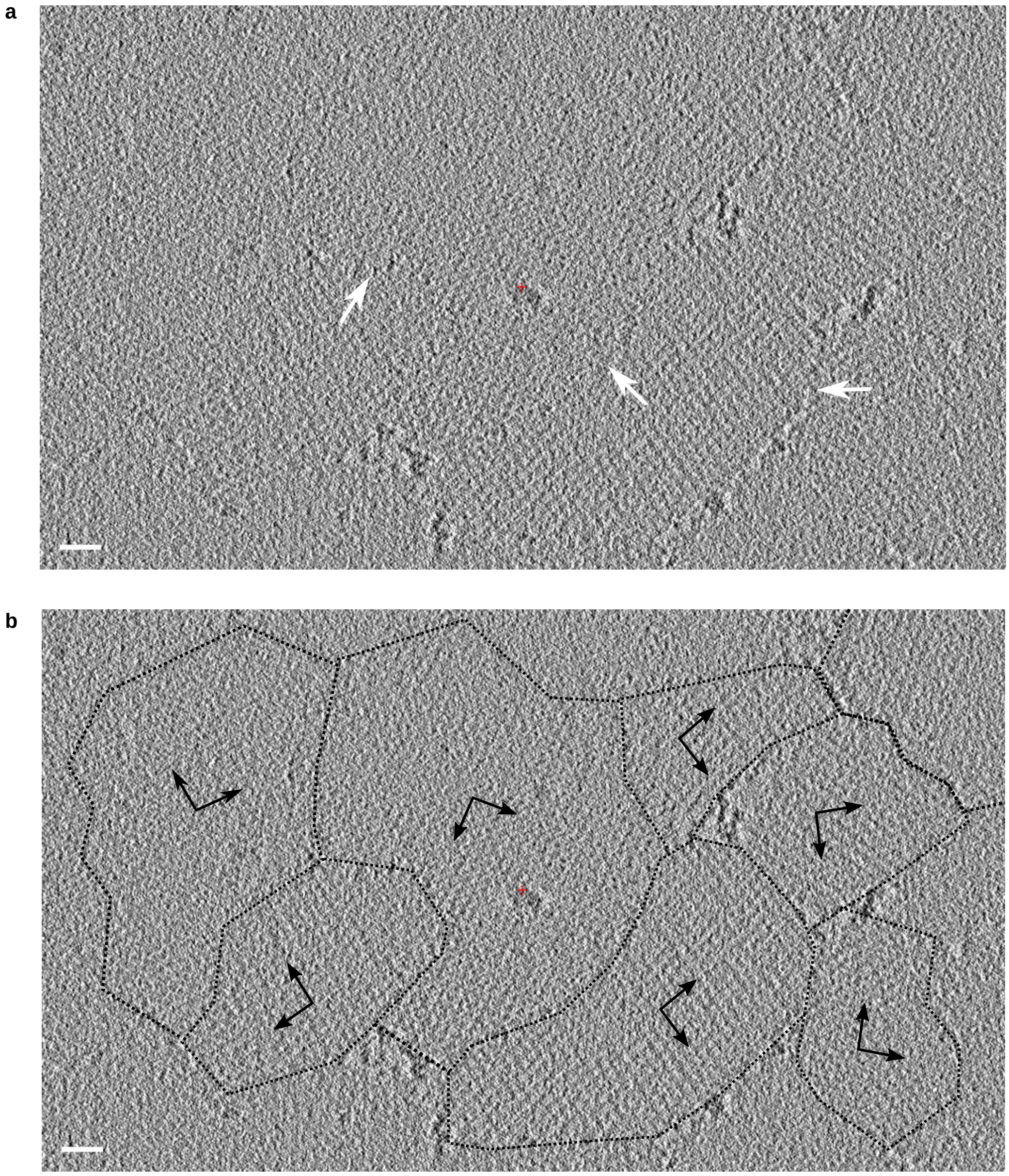

Supplementary Fig. 9: Patches of S-layer present in cryo-electron tomographic slice of extracted S-layer ghosts

a, Distinct S-layer lattice patches can be seen in tomographic slices of S-layer ghosts, with 'fault lines' present where patches intersect (white arrows). Tomogram representative of 52 tomograms collected.

b, Annotated patches from **a** (dotted lines) have distinct orientations on the surface, with unit cell axes of the different lattices highlighted (black arrows). Scale bar: 25 nm.

Supplementary Table 1 - **Data collection and refinement statistics**

	LID/HID	SLP_L/HID	H/L R7404 S-SAD	H/L R7404[1]	H/L CD630	H/L RΔD2
**Data collection**						
Space group	*C*2	*C*2	$P2_1$	$P2_1$	*P*1	*P*1
Cell dimensions						
a, *b*, *c* (Å)	73.3, 56.7, 61.8	172.9, 29.5, 144.3	76.7, 134.7, 83.9	78.1, 137.9, 84.7	72.7, 78.3, 81.6	52.8, 80.4, 81.9
α, β, γ (°)	90.0, 122.7, 90.0	90.0, 94.2, 90.0	90.0, 100.8, 90.0	90.0, 100.7, 90.0	81.9, 67.0, 65.3	97.0, 90.2, 90.2
Wavelength (Å)	0.975	0.969	2.755	0.928	0.969	0.969
Resolution (Å)	41.74- 1.50 (1.55-1.50)	53.35-2.40 (2.49-2.40)	47.70-3.00 (3.16-3.00)	83.26-2.65 (2.75-2.65)	52.31-2.55 (2.64-2.55)	44.34-3.50 (3.63-3.50)
I/σI	8.1 (2.3)	5.8 (2.1)	16.5 (1.2)	12.1 (1.4)	7.0 (1.5)	10.0 (2.5)
CC1/2	0.998 (0.975)	0.992 (0.731)	0.998 (0.707)	0.712 (0.360)	0.992 (0.732)	0.627 (0.438)
Completeness (%)	99 (97)	100 (100)	91 (80)	100 (100)	96 (97)	99 (97)
Redundancy	3.4 (2.6)	1.9 (1.9)	6.4 (6.1)	48.0 (43.0)	3.3 (3.4)	3.4 (3.5)
Anomalous completeness			91.2 (79.7)			
Anomalous multiplicity			3.3 (3.3)			
**Refinement**						
Resolution (Å)	41.74- 1.50	53.35-2.40		83.26-2.65	52.31-2.55	44.34-3.50
No. reflections	33987	29329		51027	47200	16606
R_{work} / R_{free}	18.1/21.0	25.3/29.9		22.6/27.8	24.3/26.5	25.5/27.6
No. atoms						
Protein	1709	3477		9945	10306	7890
Ligand/ion	-	-		40	15	-
Water	107	166		111	97	-
B-factors (Å^2)						
Protein	33.1	37.4		71.9	60.2	37.0
Ligand/ion	-	-		71.8	123.3	-
Water	37.6	33.1		59.4	46.0	-
Ramachandran %						
favoured	100.0	98.4		97.2	98.5	96.1
allowed	0.0	1.6		2.6	1.5	3.8
outliers	0.0	0.00		0.2	0.00	0.1
R.m.s. deviations						
Bond lengths (Å)	0.009	0.003		0.007	0.006	0.003
Bond angles (°)	1.14	0.58		1.19	1.01	0.60
PDB ID	7ACW	7ACV		7ACX	7ACY	7ACZ

*Values in parentheses are for highest-resolution shell.

[1]Two crystals were used to determine the structure of H/L complex R7404.

Supplementary Table 2 - EM data and reconstruction statistics

	$SlpA_{R20291}$	$SlpA_{R\Delta D2}$
**3D merging statistics (EM reconstructions; negative staining)**		
Resolution limit (Å)	20	20
No. structure factors	1085	667
Overall R-factor	0.33	0.33
Overall phase residual (°)	22.3	13.9
**Phase residuals in CryoEM projections (*p*2-averaged Fourier terms)**		
No. independent phases		
Resolution shell (Å)		
∞ – 15	43	43
15 - 11	42	42
11 - 8.7	47	45
8.7 – 7.5	44	-
Mean value phase error*		
Resolution shell (Å)		
∞ – 15	15.3	21.5
15 - 11	22.9	28.7
11 - 8.7	32.2	33.8
8.7 – 7.5	36.8	-
Standard error (°)		
Resolution shell (Å)		
∞ – 15	2.4	3.5
15 - 11	2.8	4.1
11 - 8.7	3.6	3.6
8.7 – 7.5	3.9	-

*Mean value phase error against symmetry-imposed phase of 0° or 180° (45° is expected for random phases[1]).

Supplementary Table 3 – Symmetry table for cryo-EM reconstruction of $SlpA_{R20291}$

Plane Group†	Phase residual (°) (random = 90°)	Target residual‡ (°)
p1	27.0	-
p2*	37.5	39.7
p12b	77.6	29.8
p12a	55.1	29.8
p121b	57.2	29.8
p121a	51.0	29.8
c12b	77.6	29.8
c12a	55.1	29.8
p222	67.7	33.6
p2221b	52.7	33.6
p2221a	47.5	33.6
p2221a	62.2	33.6
c222	67.7	33.6
p4	46.8	33.6
p422	61.9	30.1
p4212	63.5	30.1
p3	52.5	27.0
p312	60.4	27.7
p321	63.0	28.5
p6	50.4	31.6
p622	58.1	29.3

*Represents most likely plane group
†a and b represent the respective symmetry axis for the plane group
‡Target residual indicates the expected phase residual of each symmetry group based on the signal-to-noise ratio of the respective reflections[2].

Supplementary Table 4. Bacterial strains and plasmids used in x-ray crystallography and protein-protein interaction studies.

Strain or plasmid	Description	Reference/ Source Application
***C. difficile* strains**		
CD630	Ribotype 012, SLCT-7	[3]
R20291	Ribotype 027, SLCT-4	[4]
FM2.5	R20291 *slpA* 282_283insA	[5]
RΔD2	FM2.5 *slpA*ΔD2	This study
R7404	Ribotype 017, SLCT-7b	[6]
***E. coli* strains**		
NEB5a	fhuA2 Δ(*argF-lacZ*)U169 *phoA glnV44* Φ80 Δ(*lacZ*)M15 *gyrA96 recA1 relA1 endA1 thi-1 hsdR17*	New England Biolabs
BL21 (DE3)	E. *coli* str. B F– *ompT gal dcm lon hsdSB(rB–mB–)* λ(DE3 [*lacI lacU*V5-T7p07 *ind1 sam7 nin5*]) [*mal*B+]K-12(λS)	Novagen
Rosetta (DE3)	F- *ompT hsdSB*(*rB- mB-*) *gal dcm* (DE3) pRARE (CamR)	Novagen
**Plasmids**		
pOB001	pMTL960-Ptet-*slpA*ΔD2 (R20291)	This study.<br>RF102/ RF103
pRPF170	pMTL960-Ptet-*slpA* (CD630)	This study.<br>NF1414/ NF1415
pRPF233	pMTL960-Ptet-*slpA* (R20291)	[5]
**Plasmids for recombinant expression of mature protein**		
pABS17	pET28a-SLP_L-6xHis-tag (R20291)	This study.<br>SLP_L of R20291;<br>oABS46/ oABS47
pABS18	pET28a-SLP_H-6xHis-tag (R20291)	This study. SLP_H of R20291;<br>oABS44/ oABS45
pABS19	pET28a-SLP_L-6xHis-tag (R7404)	This study. SLP_L of R7404;<br>oABS1/ oABS2

pABS20	pET28a-SLP_H-6xHis-tag (R7404)	This study. SLP_H of R7404; *Nco*I/*Xho*I subcloning from pJAK148 into pET28a
pABS21	pET28a-SLP_LΔLID-6xHis-tag (R20291)	This study. SLP_L of R20291 lacking LID; oABS31/oABS48
pABS22	pET28a-SLP_HΔHID-6xHis-tag (R20291)	This study. SLP_H of R20291 lacking HID; oABS15/oABS16
pABS23	pET28a-SLP_LΔLID-6xHis-tag (R7404)	This study. SLP_L of R7404 lacking LID; oABS31/oABS32
pABS24	pET28a-SLP_HΔHID-6xHis-tag (R7404)	This study. SLP_H of R7404 lacking HID; oABS39/oABS16
pSLPH630	pET28a-SLP_H-6xHis-tag (CD630)	SLP_H of CD630
pSLPHΔ1-40	pET28a-SLP_HΔHID-6xHis-tag (CD630)	[7]SLP_H of CD630 lacking the N-terminal HID
pSLPL630	pET28a-SLP_L-6xHis-tag (CD630)	[7]SLP_L of CD630
pSLPLΔ260-321	pET28a-SLP_LΔLID-6xHis-tag (CD630)	[7]SLP_L of CD630 lacking the C-terminal LID
pJAK149	pETDuet-1-HID-6xHis-tag –SLP_L (R7404)	This study. Recombinant co-expression of mature SLP_L/HID of R7404; RF1396/ RF1397, RF1398/ RF1400, RF1394/ RF1395
pJAK147	pETDuet-1-HID-6xHis-tag – LID (R7404)	This study. Recombinant co-expression of mature LID/HID of R7404; RF1396/ RF1397, RF1398/ RF1400, RF1395/ RF1396
**Plasmids for expression of the mature protein with a point mutation in the interaction domain, as specified in subscript**		
pABS1	pET28a-$SLP_{L\ F274A}$-6xHis-tag (CD630)	This study. oABS33/ oABS34
pABS2	pET28a-$SLP_{H\ Y27A}$-6xHis-tag (R20291)	This study. oABS37/ oABS38
pABS3	pET28a-$SLP_{L\ F273A}$-6xHis-tag (R20291)	This study. oABS35/ oABS36
pABS4	pET28a-$SLP_{H\ Y26A}$-6xHis-tag (R7404)	This study. oABS5/ oABS6

pABS5	pET28a-$SLP_{L\ F270A}$-6xHis-tag (R7404)	This study. oABS21/ oABS22
pABS6	pET28a-$SLP_{H\ N18A}$-6xHis-tag (R7404)	This study. oABS3/ oABS4
pABS7	pET28a-$SLP_{H\ D28A}$-6xHis-tag (R7404)	This study. oABS7/ oABS8
pABS9	pET28a-$SLP_{H\ N34A}$-6xHis-tag (R7404)	This study. oABS11/ oABS12
pABS11	pET28a-$SLP_{L\ D254A}$-6xHis-tag (R7404)	This study. oABS17/ oABS18
pABS12	pET28a-$SLP_{L\ I259A}$-6xHis-tag (R7404)	This study. oABS19/ oABS20
pABS13	pET28a-$SLP_{L\ Y279A}$-6xHis-tag (R7404)	This study. oABS23/ oABS24
pABS14	pET28a-$SLP_{L\ G300A}$-6xHis-tag (R7404)	This study. oABS25/ oABS26
pABS16	pET28a-$SLP_{L\ R312A}$-6xHis-tag (R7404)	This study. oABS29/ oABS30
pSLPH_Y27A	pET28a-$SLP_{H\ Y27A}$-6xHis-tag (CD630)	This study. NF1386/ NF1387
pJAK186	pMTL960 Ptet-*slpA*$SLP_{H\ Y27A}$ (CD630)	This study. NF1386/ NF1387
pRPF209	pMTL960-Ptet-*slpA*$SLP_{L\ F274A}$ (CD630)	This study. NF1189/ NF1190

Supplementary Table 5. Oligonucleotides used in this study

Name	Sequence	Application
oABS1	GATCCCATGGCAGATAGTAC	Amplification of SLP_L R7404 with *Nco*I site forward primer
oABS2	GATCCTCGAGAGATTTAGTTTC	Amplification of SLP_L R7404 with *Xho*I site reverse primer
oABS3	GCTAAATTAAAAGATTTAAAAGATTATGTAG	Introduction of N18A point mutation in R7404 SLP_H
oABS4	AGCTTTTATAGTTATTTTAGCTGG	Introduction of N18A point mutation in R7404 SLP_H
oABS5	GCTGTAGATGATTTAAAAACATAC	Introduction of Y26A point mutation in R7404 SLP_H
oABS6	ATCTTTTAAATCTTTTAATTTATTAGC	Introduction of Y26A point mutation in R7404 SLP_H
oABS7	GCTGATTTAAAAACATACAATAATAC	Introduction of D28A point mutation in R7404 SLP_H
oABS8	TACATAATCTTTTAAATCTTTTAATTTATTAG	Introduction of D28A point mutation in R7404 SLP_H
oABS11	GCTAATACTTACTCAAATGTTGTAAC	Introduction of N34A point mutation in R7404 SLP_H
oABS12	GTATGTTTTTAAATCATCTACATAATC	Introduction of N34A point mutation in R7404 SLP_H
oABS15	GCGCGCACAGTAGCAGGAGAAGATAGAATAG	Deletion of HID in R7404 SLP_H
oABS16	CATGGTATATCTCCTTCTTAAAGTTAAAC	Deletion of HID in SLP_H (R7404, R20291)
oABS17	GCTTCAAGTTCATATATTAGTGC	Introduction of D254A point mutation in R7404 SLP_L
oABS18	CACATCAATAGATTCTTCTTTTGC	Introduction of D254A point mutation in R7404 SLP_L
oABS19	GCTAGTGCTGAAAATTTAGC	Introduction of I259A point mutation in R7404 SLP_L
oABS20	ATATGAACTTGAATCCACATC	Introduction of I259A point mutation in R7404 SLP_L
oABS21	GCTAATCCTAAAGAGGTTTCTG	Introduction of F270A point mutation in R7404 SLP_L
oABS22	TACATATTTTTTAGCTAAATTTTCAGCAC	Introduction of F270A point mutation in R7404 SLP_L
oABS23	GCTAATGCAATAGTTGCATTAC	Introduction of Y279A point mutation in R7404 SLP_L

oABS24	AGCTTCAGAAACCTCTTTAGG	Introduction of Y279A point mutation in R7404 SLP_L
oABS25	GCAAAATATCAAGTTATTTTCTATCC	Introduction of G300A point mutation in R7404 SLP_L
oABS25	ATTAACTAATTGTACTAAATCAGATTC	Introduction of G300A point mutation in R7404 SLP_L
oABS29	GCATTAGAAACTAAATCTCTCG	Introduction of R312A point mutation in R7404 SLP_L
oABS30	TTTTCCTTCTGGATAGAAAATAAC	Introduction of R312A point mutation in R7404 SLP_L
oABS31	GCGCGCCTCGAGCACCACCACCAC	Deletion of LID in SLP_L (R7404, R20291)
oABS32	TTTTATAGTACCTGTTGCAGCCATATC	Deletion of LID in R7404 SLP_L
oABS33	GCTGATCCAGATGAAATTTCTG	Introduction of F274A point mutation in CD630 SLP_L
oABS34	TACATATCTTTTAGCTAAATTTTCAGC	Introduction of F274A point mutation in CD630 SLP_L
oABS44	GATCCCATGGCTGCAAAGGCTTCAATTGCTG	Amplification of SLP_H R20291 with *Nco*I site forward primer
oABS45	GATCCTCGAGCATACTTAATAAATCTTTT AATTTATTTATAACTG	Amplification of SLP_H R20291 with *Xho*I site reverse primer
oABS46	GATCCATGGCAGAAGATATGTCGAAAGTTG	Amplification of SLP_L R20291 with 5' *Nco*I site
oABS47	GATCCTCGAGACTCTTAGTTGTAACTCTTTTTCC	Amplification of SLP_L R20291 with 3' *Xho*I site
oABS48	AGTTATTACTGGGCTTCCAGATTGTG	Introduction of deletion of LID in R20291 SLP_L
oABS35	GCTAATAAAACAGATTTAAATACTCTTTAC	Introduction of F273A point mutation in R20291 SLP_L
oABS36	TACATATTTTTTAGCTAAATCTTGTGCTG	Introduction of F273A point mutation in R20291 SLP_L
oABS37	GCTGTGGATGATTTAAGAACATATAATAATG	Introduction of Y27A point mutation in R20291 SLP_H
oABS38	ATCTTTTAAGTCTTTCTTCTTATCTGAC	Introduction of Y27A point mutation in R20291 SLP_H
oABS39	GCGCGCGAAGTAGCAGGAGAAGATAG	Deletion of HID in R20291 SLP_H
NF1189	GCAGATCCAGATGAAATTTCTGAAGC	Introduction of F274A point mutation in CD630 SLP_L

NF1190	TACATATCTTTTAGCTAAATTTTCAGCTG	Introduction of F274A point mutation in CD630 SLP_L
NF1386	GCAGTAGATGATTTAAAAACATATAATAATACTTATTC	Introduction of Y27A point mutation in CD630 SLP_H
NF1387	ATCTTTTAAATCTTTTAATTTATTAGCTTTTATAAC	Introduction of Y27A point mutation in CD630 SLP_H
NF1414	GATCGAGCTCTATAATGTTGGGAGGAATTTAAGAAATG	Amplification of *slpA* from CD630 with 5' *Sac*I site
NF1415	GATCGGATCCTTACATATCTAATAAATCTTTCATTTTG	Amplification of *slpA* from CD630 with 3' *Bam*HI site
RF102	GGTTCTGGAAGCCCAGTAATAACTAAAC	Replacement of coding sequence of SLP_L domain 2 with GGA GGT
RF103	TCCAGAGCTTATTAAGAAATCTACATAATCC	Replacement of coding sequence of SLP_L domain 2 with GGA GGT
RF1393	GATCCATATGGCAGATAGTACTACGCCAGG	Amplification of SLP_L for insertion into pACYC-Duet1
RF1394	GATCGGTACCTTAAGATTTAGTTTCTAATCTTTTTCCTTCTG	Amplification of LID and SLP_L for insertion into pACYC-Duet1
RF1395	GATCCATATGGTTAGAGTTACAAGTGCAAAAGAAG	Amplification of LID for insertion into pACYC-Duet1
RF1396	CATGGTATATCTCCTTATTAAAGTTAAAC	Linearization of pACYC-Duet1 for insertion of HID
RF1397	CTCGAGCACCACCACCAC	Linearization of pACYC-Duet1 for insertion of HID
RF1398	TGTTTAACTTTAATAAGGAGATATACCATGGCAGATATAATAGCTGATGCAG	Amplification of HID for insertion into pACYC-Duet1
RF1400	ATCTCAGTGGTGGTGGTGGTGGTGCTCGAGTACAACATTTGAGTAAGTATTATTGTATG	Amplification of HID for insertion into pACYC-Duet1

SUPPLEMENTARY REFERENCES

1. Bullough, P. A. & Henderson, R. Phase accuracy in high-resolution electron microscopy of trigonal and orthorhombic purple membrane. *Biophys. J.* **58**, 705 (1990).
2. Valpuesta, J. M., Carrascosa, J. L. & Henderson, R. Analysis of electron microscope images and electron diffraction patterns of thin crystals of Ø29 connectors in ice. *Journal of Molecular Biology* **240**, 281–287 (1994).
3. Wust, J., Sullivan, N. M., Hardegger, U. & Wilkins, T. D. Investigation of an outbreak of antibiotic-associated colitis by various typing methods. *J. Clin. Microbiol.* **16**, 1096–1101 (1982).
4. Stabler, R. A. *et al.* Comparative genome and phenotypic analysis of *Clostridium difficile* 027 strains provides insight into the evolution of a hypervirulent bacterium. *Genome Biol.* **10**, R102 (2009).
5. Kirk, J. A. *et al.* New class of precision antimicrobials redefines role of *Clostridium difficile* S-layer in virulence and viability. *Sci. Transl. Med.* **9**, eaah6813 (2017).
6. Stubbs, S. L. J., Brazier, J. S., O'Neill, G. L. & Duerden, B. I. PCR targeted to the 16S-23S rRNA gene intergenic spacer region of *Clostridium difficile* and construction of a library consisting of 116 different PCR ribotypes. *J. Clin. Microbiol.* **37**, 461–463 (1999).
7. Fagan, R. P. *et al.* Structural insights into the molecular organization of the S-layer from *Clostridium difficile*. *Mol. Microbiol.* **71**, 1308–1322 (2009).
