# Supplementary material for: Structure and assembly of the S-layer in C. difficile
Source: Nat Commun. 2022 Feb 25;13:970. doi: 10.1038/s41467-022-28196-w (PMC8881574; doi:10.1038/s41467-022-28196-w)
Supplement: Supplementary file 2 — Description of Additional Supplementary Files [file 41467_2022_28196_MOESM2_ESM.pdf]

Description of Additional Supplementary Files

File Name: Supplementary Movie 1

Description: **S-layer structure and assembly**

Structure of the main S-layer component in C. difficile, the SLPH/SLPL complex determined by X-ray crystallography and how it fits the reconstruction of the S-layer in situ in the cell. The compact assembly is shown, highlighting the key pores in the lattice. Removal of the more exposed domain in SLPL (D2) doesn‟t affect overall structure or assembly but seems to generate a more porous S-layer. Representations are as defined in Figures 1, 3 and 4.
