# Supplementary material for: Structure and assembly of the S-layer in C. difficile
Source: Nat Commun. 2022 Feb 25;13:970. doi: 10.1038/s41467-022-28196-w (PMC8881574; doi:10.1038/s41467-022-28196-w)
Supplement: Supplementary file 4 — Reporting summary [file 41467_2022_28196_MOESM4_ESM.pdf]

nature portfolio

Corresponding author(s): Paula S. Salgado
Robert P. Fagan
Per A. Bullough

Last updated by author(s): Dec 7, 2021

Reporting Summary

Nature Portfolio wishes to improve the reproducibility of the work that we publish. This form provides structure for consistency and transparency in reporting. For further information on Nature Portfolio policies, see our Editorial Policies and the Editorial Policy Checklist.

Statistics

For all statistical analyses, confirm that the following items are present in the figure legend, table legend, main text, or Methods section.

n/a	Confirmed	
☐	☒	The exact sample size (*n*) for each experimental group/condition, given as a discrete number and unit of measurement
☐	☒	A statement on whether measurements were taken from distinct samples or whether the same sample was measured repeatedly
☐	☒	The statistical test(s) used AND whether they are one- or two-sided<br>*Only common tests should be described solely by name; describe more complex techniques in the Methods section.*
☒	☐	A description of all covariates tested
☒	☐	A description of any assumptions or corrections, such as tests of normality and adjustment for multiple comparisons
☐	☒	A full description of the statistical parameters including central tendency (e.g. means) or other basic estimates (e.g. regression coefficient) AND variation (e.g. standard deviation) or associated estimates of uncertainty (e.g. confidence intervals)
☒	☐	For null hypothesis testing, the test statistic (e.g. *F*, *t*, *r*) with confidence intervals, effect sizes, degrees of freedom and *P* value noted<br>*Give P values as exact values whenever suitable.*
☒	☐	For Bayesian analysis, information on the choice of priors and Markov chain Monte Carlo settings
☒	☐	For hierarchical and complex designs, identification of the appropriate level for tests and full reporting of outcomes
☒	☐	Estimates of effect sizes (e.g. Cohen's *d*, Pearson's *r*), indicating how they were calculated

Our web collection on statistics for biologists contains articles on many of the points above.

Software and code

Policy information about availability of computer code

Data collection	X-ray data collection software: Xia2 v0.5.653, ISPyB v4.11.0<br>Tomography data collection: SerialEM (used Jan 2019)
Data analysis	X-ray crystallography data processing and analysis software: XDS v20180126, iMosflm v7.2.2, DIALS v1.5.1, Aimless v0.5.32, CCP4i v.7.0.65, CCP4i2 v1.1.00, PARROT v1.0.5, Arcimboldo_lite (within CCP4 v7.0.065), Buccaneer v1.6.4, Coot 0.8.9, Phenix v1.19.2, Phaser v2.8.2, Anode 2013/1, CheckMyBlob webserver (access Aug 2020), REFMAC 5.8.0258, PDB-REDO v7.3 , MOLPROBITY v4.5.1, ISOLDE v1.1.0<br>Electron crystallography data processing software: CCP4i v. 7.0.078, 2dx v3.4.2 which includes QUADSEARCH, CCUNBEND, ALLSPACE, ORIGTILTK, LATLINE and SCALIMAMP3D<br>Structure visualisation tools: Chimera 1.13.1, PyMOL v2.5.0<br>Tomography analysis: IMOD v4.9.11<br>Bioinformatic/structural analysis: PDBeFold v2.59, PISA 2.0.9, PDBSum webserver (access Oct 2019), LigPLot+ v2, DynDom6D v1.0, HingeProt webserver (access March 2020), DynOmics , ConSurf v3 webserver (access Jan 2020), ProtTest 3.4.1, MAFFT v.7, ALINE v011208, SWISS-MODEL webserver (access May 2020), Numpy v1.16.6, Pandas v0.24.2, Seaborn v0.10.1, ESPript3 v3.07, ChexVis webserver (access Aug2020)<br>Statistical analysis: GraphPad Prism 8

For manuscripts utilizing custom algorithms or software that are central to the research but not yet described in published literature, software must be made available to editors and reviewers. We strongly encourage code deposition in a community repository (e.g. GitHub). See the Nature Portfolio guidelines for submitting code & software for further information.

Data

Policy information about availability of data

All manuscripts must include a data availability statement. This statement should provide the following information, where applicable:

- Accession codes, unique identifiers, or web links for publicly available datasets
- A description of any restrictions on data availability
- For clinical datasets or third party data, please ensure that the statement adheres to our policy

The datasets generated during and/or analysed during the current study are available from the corresponding authors on reasonable request.
X-ray structural data is publicly available via the PDB repository under PDB IDs: 7ACW (https://doi.org/10.2210/pdb7ACW/pdb), 7ACV (https://doi.org/10.2210/pdb7ACV/pdb), 7ACX (https://doi.org/10.2210/pdb7ACX/pdb), 7ACY (https://doi.org/10.2210/pdb7ACY/pdb), 7ACZ (https://doi.org/10.2210/pdb7ACZ/pdb)
Electron crystallography data will be available from the authors upon reasonable requestare available in the EMDB repository under accession code EMD-13957 and the fitted model is available in PDB repository with PDB ID: 7QGQ.

Field-specific reporting

Please select the one below that is the best fit for your research. If you are not sure, read the appropriate sections before making your selection.

☒ Life sciences ☐ Behavioural & social sciences ☐ Ecological, evolutionary & environmental sciences

For a reference copy of the document with all sections, see nature.com/documents/nr-reporting-summary-flat.pdf

Life sciences study design

All studies must disclose on these points even when the disclosure is negative.

Sample size	For all experiments, sample size was determined by experimental design and appropriate replicates carried out. The variants tested in ELISA assays were selected based on X-ray structural models and the interactions between different domains. For lysozyme resistance assays, the mutant and wild type strains were selected, sampled at regular hourly intervals during growth to include all phases of growth, as standard.
Data exclusions	No data was excluded
Replication	Experiments were carried out in biological and/or technical replicates, as detailed in the methods. For ELISA assays, experiments were carried out in technical triplicates for each combination of the variants tested. Lysozyme resistance assays were carried in technical triplicates of two independent biological replicates of the 3 strains to be analysed.
Randomization	Randomization is generally applicable to clinical trials or animal experiments. All studies reported were in vitro, with biological replication of bacterial samples reducing any potential bias or systematic variation.
Blinding	Blinding is generally applicable to clinical trials or animal experiments. All studies reported were in vitro, with biological replication of bacterial samples reducing any potential bias or systematic variation.

Reporting for specific materials, systems and methods

We require information from authors about some types of materials, experimental systems and methods used in many studies. Here, indicate whether each material, system or method listed is relevant to your study. If you are not sure if a list item applies to your research, read the appropriate section before selecting a response.

Materials & experimental systems

n/a	Involved in the study	
☐	☒	Antibodies
☒	☐	Eukaryotic cell lines
☒	☐	Palaeontology and archaeology
☒	☐	Animals and other organisms
☒	☐	Human research participants
☒	☐	Clinical data
☒	☐	Dual use research of concern

Methods

n/a	Involved in the study	
☒	☐	ChIP-seq
☒	☐	Flow cytometry
☒	☐	MRI-based neuroimaging

Antibodies

Antibodies used	polyclonal rabbit primary antibodies anti-SLPL and anti-SLPH (Fagan et al. Mol. Microb, 2009); HRP-conjugated secondary anti-rabbit antibody (ELISA - Promega, W4011 and western immunoblotting - Invitrogen)

Validation	Anti-SLPL and anti-SLPH antibodies validation described in Fagan et al., Mol Microb, 2009.<br>HRP-conjugated secondary anti-rabbit antibody validation as per manufacturer details
